# Statistical analysis of 3D localisation microscopy images for quantification of membrane protein distributions in a platelet clot model

**DOI:** 10.1371/journal.pcbi.1007902

**Published:** 2020-06-30

**Authors:** Sandra Mayr, Fabian Hauser, Sujitha Puthukodan, Markus Axmann, Janett Göhring, Jaroslaw Jacak

**Affiliations:** 1 University of Applied Sciences Upper Austria, Linz, Austria; 2 Johannes Kepler University Linz, Linz, Austria; 3 Center for Pathophysiology, Infectiology and Immunology, Institute for Hygiene and Applied Immunology, Medical University of Vienna, Vienna, Austria; University of Cambridge, UNITED KINGDOM

## Abstract

We present the software platform 2CALM that allows for a comparative analysis of 3D localisation microscopy data representing protein distributions in two biological samples. The in-depth statistical analysis reveals differences between samples at the nanoscopic level using parameters such as cluster-density and -curvature. An automatic classification system combines multiplex and multi-level statistical approaches into one comprehensive parameter for similarity testing of the compared samples. We demonstrated the biological importance of 2CALM, comparing the protein distributions of CD41 and CD62p on activated platelets in a 3D artificial clot. Additionally, using 2CALM, we quantified the impact of the inflammatory cytokine interleukin-1β on platelet activation in clots. The platform is applicable to any other cell type and biological system and can provide new insights into biological and medical applications.

## Author summary

Single-molecule localisation microscopy (LM) became more accessible over the past years. Companies and research facilities developed instruments for 3D/2D LM, but there is a lack of comparison methods for LM-images. Our tool offers a new comparative analysis of LM data. Our system is capable of showing the difference on the level of single molecule clusters and provides information on differences between images on various scales. We determine if clusters formed of molecule localisations are comparable. We display the difference on density or the shape of these formed clusters for a certain dimension. Since the comparison is performed for all dimensions, the differences between cluster properties are observed very precisely. Thereby, we learn if clusters of molecules are formed and how they differ in both samples. For a fast/concluding comparison, we have added tools which derive a percentage of equality of both images based on all comparison results. The tool is useful for comparison of images of molecules in cells, which are expected to differ from each other (exemplified with platelets). The differences might be caused by drug treatment, different disease progress or other environmental/genetical issues. The analysis is performed at a single cell level as well as cell cluster level.

This is a *PLOS Computational Biology* Methods paper.

## Introduction

LM has progressed immensely over the last decade [1–5], however only a few of the approaches towards a comparative analysis of the resulting data have been achieved [6–9]. Primarily, these studies utilised comparative analyses of single molecules in the context of co-localisation in cells [10–14], with the majority visualising the 2D and 3D arrangement of proteins [10, 12, 13, 15, 16].

First attempts for cluster analysis for LM data, developed by Owen et al. [17], were based on Ripley's K-function, which quantifies the global distribution and heterogeneity of proteins at the cell plasma membrane. Alternatively, analysis systems like SR-Tesseler [18] or ClusterVisu [19] based on Voronoi tessellation were developed. These methods were well suited for visualization and rendering of localisation density distribution in a sample or colocalization between molecules primarily for 2D data.

Clustering–the formation of micro- and nano-domains within plasma membranes–is now a widely recognized feature that ensures hierarchical organisation of many proteins. The functions of these clusters are diverse [20, 21] and impaired integrin clustering for example has been shown to be involved in thrombasthenia [22]. However, there is a general lack of methods that enable a comparative analysis of localisation microscopy data on protein distributions and clustering.

Mainly, the presented analysis extracts spatial descriptors (spatial features), which allow the determination of the similarity of 3D localisation microscopy data of two samples, regardless of their rotation, translation and quantity. The basis for the feature extraction is multiple resampling of both samples with a given number of localisations (typically between 2 000 and 10 000) which increases the amount of data for statistical analysis and reduces the calculation time to minutes for very large samples (> 200 000 points = localisations). Furthermore, our method does not require transformation of the coordinate system, synchronisation of the region of interest (ROI) size or possible normalization for both samples.

For each pair of such bootstrap sub-samples, sequential hierarchical clustering of localisations is performed. To characterise clusters with a specific size, two crucial parameters have been derived–density and curvature distribution. For these distributions we perform nonparametric 2-sample statistical tests (such as Kolmogorov-Smirnov test, Wilcoxon rank sum test) and build a p-value-map (for each pair of samples and each cluster size, forming a first level of analysis). These p-value maps are directly used among other parameters (aggregated p-values) from second level of analysis to train the multilayer perceptron (MLP) neural network. In addition, in order to build a more robust analytical measure of similarity, we average over the generated distributions and their aggregations and generalizations. To aggregate the results of the 2-sample statistical tests, the weighted AND operator of fuzzy membership functions is used. The generalisation is performed by applying statistical tests on average values of density and curvature distributions and on values of the Ripley’s K-function. The maximum Ripley’s H function is used primarily to establish the parameters for the random sample generator.

Currently, global comparative analyses of clot formations have only used diffraction-limited fluorescence microscopy to characterise morphological changes (e.g. shape change upon activation) and the cytoskeletal organisation of platelets (e.g. actin and tubulin reorganisation) [23–29]. Nanoscopic localisation microscopy analyses have only been performed for single platelets characterising the content of α-granules [30], the arrangement of actin filaments [31, 32], various actin associated proteins (e.g. P4 or vinculin), and mitochondria [33, 34]. Therefore, an artificially formed clot presents a proficient system to characterise environmentally-induced protein redistributions on a nanoscopic scale.

In this study, we present a software tool that quantitatively compares and classifies two biological samples based on the protein distributions at the nanoscale using 3D localisation microscopy data. Our tool– 2-sample Comparative Analysis of 3D Localisation Microscopy Data (2CALM)–is an analysis pipeline, which organizes LM data into protein clusters of different dimensions and calculates the samples’ statistical parameters using various numerical methods. The images obtained from LM can be regarded as a 3D cloud of points. A comparative analysis of such clouds requires the extraction of features representing their geometrical structure without losing accuracy. Several deep machine-learning algorithms, especially convolution neural networks (CNN), have been applied to single 3D point cloud analysis. A common approach is to rasterize the 3D point data into a 3D voxel grid [35–37]. This approach, however, suffers from a trade-off between its computational cost and its approximation accuracy. Thus, we employ a different concept and propose a new representation of a LM-derived cloud of points. This representation is based on empirical distributions of the density and the curvature of the point’s clusters within point-clouds having a predetermined maximal dimension, yielding density and curvature distributions for each sample. These distributions are compared with non-parametrical statistical tests such as Kolmogorov-Smirnov or Wilcoxon test. The results of the tests create a features-array of the similarity of both samples that is not dependent on their size and location in space. Our system uses the constructed feature-arrays to determine the analytical similarity measure between samples and to train the fully-integrated MLP neural network for automatic classification of samples similarities. Generally, the system can be used to compare any 3D and 2D cloud of points regardless the origin of the samples and primarily provides a comparison between two LM images. The results directly show the comparison between the densities/curvatures of molecular clusters and do not directly provide information’s on cellular structures.

For demonstration of the biological importance and applicability of 2CALM, we quantitatively analysed the nanoscopic distribution of CD41 and CD62p proteins on activated platelets within an artificial clot using 3D dSTORM. As changes in protein distribution during platelet activation and thrombus formation can impact further downstream signalling, the effect of the pro-inflammatory molecule interleukin 1-beta (IL-1β) on the CD62p distribution in platelets within an artificial clot was analysed.

CD41 (integrin α-IIb) is a protein that is present in the cell membrane as well as in the α-granules of platelets [38]. As part of the fibrinogen receptor GPIIb/IIIa it binds fibrinogen and von Willebrand factor (vWF). Upon activation, CD41 molecules from the granules are incorporated into the cell membrane. Platelet aggregates, which are formed by GPIIb/IIIa-fibrinogen interaction, are stabilized by the interaction of CD62p (P-selectin), which binds P-selectin glycoprotein ligand-1 and platelet sulfatides [39–41]. CD62p is a cell adhesion molecule and is transported upon activation from its location in the α-granules to the cell membrane [26, 42–46]. Activation of the coagulation system (i.e. clot formation) is heavily influenced by pro-inflammatory molecules such as IL-1β. These molecules have been shown to increase the response of the platelets towards elevated aggregation [47, 48].

The performance of 2CALM was tested by analysing the distributions of two protein types (CD41 and CD62p), which are known to exhibit different clustering behaviour upon activation. The detailed analysis of 2CALM shows the protein distributions of CD41 and CD62p at the nanoscopic level. Furthermore, 2CALM was tested on a system, which changes the protein (CD62p) distribution upon external treatment. We show that CD62p distributes diversely within untreated and IL-1β-treated clots providing different clustering-based statistical parameters. The data obtained from LM experiments on CD62p distribution in platelets were compared to simulated datasets. The presented experimental and simulated data showcase the software´s utility to support researchers in advanced two-sample comparisons of heterogeneous LM data. It will therefore advance the thorough investigation and ultimately the understanding of the nanoscopic organization of proteins in complex tissues.

## Results

**Fig 1:**
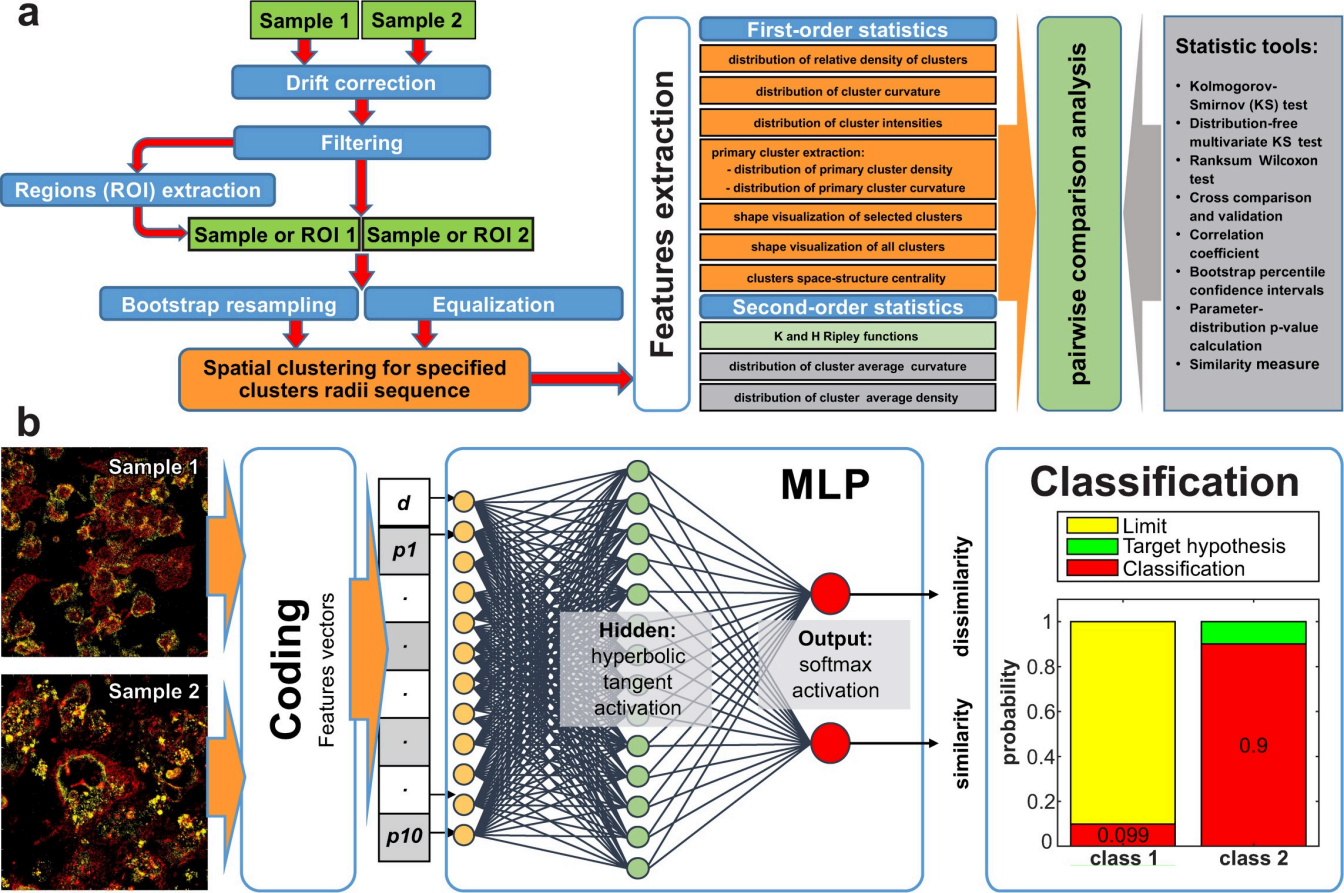
2CALM workflow. (a) represents the features of the 2CALM platform. Datasets are pre-processed using drift correction and filtering of outliers (optionally: ROI-extraction). For a full image comparison, dataset sizes are optionally equalized. The default bootstrapping step re-samples the datasets into sub-samples (used for spatial clustering). Hierarchical spatial clustering enables the extraction of the density-distribution and the curvature-distribution of clusters. For comparison, a two-level, multi-parameter analysis of different clustering dimensions is performed. Kolmogorov-Smirnov-, Wilcoxon-test and bootstrap confidence interval analysis is used for a comparison of protein distributions. (b) depicts the structure of the multilayer perceptron (MLP) neural network based machine learning module. The clustering from (a) provides parameters (feature vectors) which are used to train a MLP (including one hidden layer) network to assign the datasets to their classes (similarity classification).

We developed a software system capable of a two-sample comparison named 2CALM (MatLab environment). In several experiments, artificial clots were stained, imaged using 3D dSTORM, and analysed. The resulting analysis is comprised of information on the sub-diffractional as well as on the bulk level, obtained in one measurement. The features of the 2CALM software system are depicted in Fig 1A. This platform contains a two-level statistical analysis of 3D spatial cluster features. The 1^st^-level analysis directly compares the properties of spatial clusters (cluster density and/or cluster curvature distributions) derived from LM datasets with different clustering dimensions [49–52]. The 2^nd^-level statistical tests are performed on averaged cluster features to assess their randomness and the distribution of the average cluster’s curvature (shape) and density. The obtained sample features can then be compared pairwise using the methods of a statistic tools module, relying on a comparison of averaged statistical values (e.g. average cluster density or curvature distributions) of bootstrapped re-samples. The determined features are based on the relative cluster’s density distributions for each clustering dimension and their individual curvature distribution and are independent of cluster positions and orientations. A detailed workflow of the features extraction and aggregation is depicted in S2 Fig.

### Biological test system and image acquisition

In order to show the applicability of 2CALM, we designed an artificial clot [53]. After mixing thrombin, fibrinogen and platelets, the cells coagulate on a glass slide, which yields a viscous 3D clot. For visualisation purposes, we immuno-stained platelets within the protein matrix with fluorescently labelled antibodies that target CD41 and CD62p proteins. In all clot experiments, CD41 was labelled with Alexa488-conjugated anti-CD41-antibody, and CD62p was labelled with Alexa647-conjugated anti-CD62p-antibody. For imaging, we applied direct stochastic optical reconstruction microscopy (dSTORM) [54]. We used an oxygen scavenger system (OxEA) [55], which allows for two-colour imaging without buffer exchange. To adjust the blinking rates of both fluorophores, an additional UV-laser illumination was utilized. A cylindrical lens in the optical detection pathway of the microscope introduced astigmatism and an axially dependent deformation of the point spread function (PSF) of individual emitters. The single-molecule positions were determined using customized software with fitting routines derived from rapidSTORM [56]. Automated tools for extraction, characterization and comparison of the protein distributions at the nanoscopic level were implemented.

### Software features: Dataset pre-processing module

2CALM includes a collection of software tools enabling a pairwise comparative analysis of independent sets of localisation microscopy data. Datasets usually have a different number of localisation events (often gathered into 3D point-clouds), varying noise due to unspecific binding and/or localisation errors resulting from sample drift during the measurement. Our toolset enables noise filtration and drift correction. Typically, special convolution algorithms [57, 58] or fiducial markers [59, 60] are deployed to correct for sample drift; we expanded a developed drift correction algorithm [32] to support 3D position accuracy of localisation events [61]. The 3D dSTORM images contain single localisation events which are not of interest in cluster analysis and henceforth are classified as outlier points. Filtering of individual outlier points (or small group of points) is carried out using a DBSCAN (Density-Based Spatial Clustering of Applications with Noise) algorithm [62–65]. An additional modified version of DBSCAN [66] allows for an automatic determination of the ROI. It is used for spatially well-separated domains with single-molecule localisations (e.g. signals in sparsely distributed individual cells) (S11 Fig). Both tools–drift correction and outlier filtering–are combined in a dataset pre-processing module.

### Software features: Equalization of data sets (bootstrapping)

Although an analysis of all points in a sample at once is possible, it is rather time-consuming and less robust to errors. When datasets have a small number of localisation events (up to 50 000), a 1^st^-level statistical analysis–after equalization of both samples–can be performed. The number of points is equalized to the smaller of the pairwise compared data sets. Representative datasets are shown in S1A and S1B Fig. When larger differences in localisation events are detected, errors due to a random selection of points from the equalized sample dataset may lead to an incorrect comparison, even though the samples originate from the same biological replica. In such instances bootstrapping is used to randomly draw events from the measured datasets, which yields an equalized data set. In order to improve the robustness of the analysis, we performed multiple re-sampling (bootstrapping) [67–69] using a defined number of points. The bootstrap procedure involves choosing random samples (with replacement) from a large dataset and analysing each bootstrap sample in a similar way. Bootstrapping reduces the risk of accidental one-time errors (when there are not sufficient points in a subgroup) and allows for better parameter-estimation for subsequent comparative analyses.

### Software features: Statistical analysis of cluster features (Two-level statistics)

The 1^st^ -level analysis relies on a direct comparison of sample features like cluster density and cluster curvature distributions for each cluster dimension. For a comparison between the protein distributions of the two samples, non-parametric statistical tests are used. The 2^nd^-level analysis combines the parameters obtained from the 1^st^-level analysis and compares them globally. It relies on a comparison of averaged values of localisation densities and curvatures per cluster dimension and eliminates the influence of noise (disturbance of point localisations). Additional K-/H-Ripley’s tests [70, 71] show the deviation of the distributions from a random Poisson distribution.

### Software features: Classification of similarity (Two-sample comparison)

#### Statistical Tools

Various statistical tests are implemented for a holistic two-sample comparison at nanoscopic and microscopic level. Non-parametric Kolmogorov-Smirnov (KS) [72–74] and Wilcoxon (WX) [75, 76] tests were used for a pairwise comparison of cluster features. In order to calculate the aggregated p-value (e.g. fusion of results of KS and WX tests) we applied the weighted t-norm-AND operator [77, 78] on the p-values of the KS-/WX-test obtained from the bootstrapping process. By introducing appropriate weights for the t-norm-AND operators the significance of a particular test increases. These tests precisely identify the cluster parameters necessary for classification of sample similarity/dissimilarity for any clustering dimension. It is not capable to automatically classify data sets.

**Fig 2:**
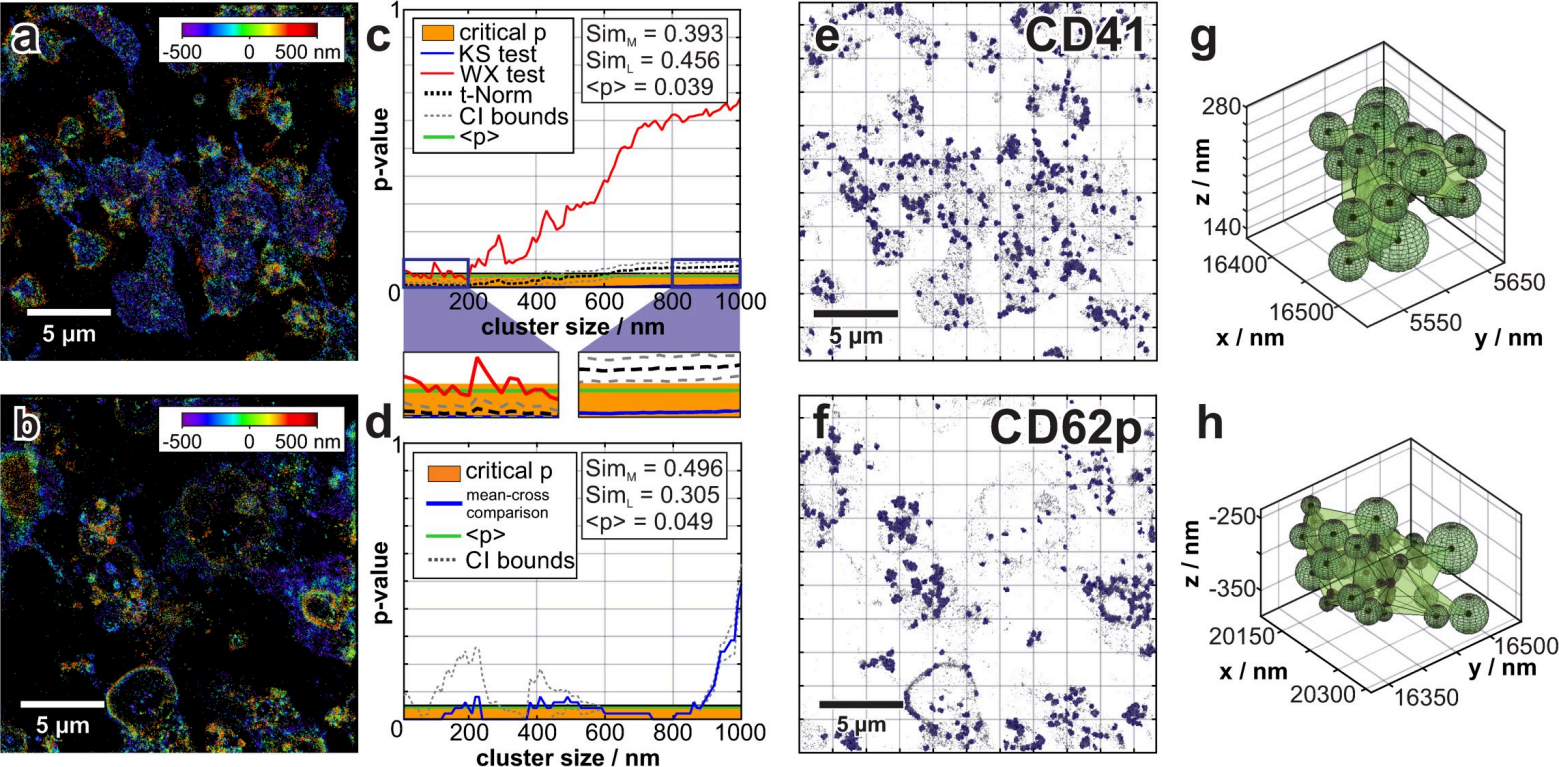
Two sample comparison of the CD62p and CD41 distributions in clots. (a) and (b) show reconstructed 3D dSTORM images (approx. 100 000 data points/image). (a) shows the 3D distribution of CD41 (Alexa488-antibody). (b) represents the 3D distribution of CD62p (Alexa647-antibody). (c) shows the comparison of the cluster densities for all given cluster dimensions between the two datasets (1^st^-level comparison). The blue/red lines depict the KS- and WX-test results, respectively. The aggregated p-value for KS and WX tests (dashed black line) remains within the critical p-value-area (orange) disproving the similarity hypothesis. The zoomed-in areas depict: a cluster dimension (5 nm—200 nm, *sim*_*M*_, *sim*_*L*_ below 0.5); pairwise KS- and WX-test comparisons of cluster densities for both samples indicate dissimilarity (left) and a second cluster dimension (800 nm—1000 nm, *sim*_*M*_, *sim*_*L*_ larger 0.5); pairwise KS- and WX-test comparisons of cluster densities for both samples show similarity (right). (d) shows the 2^nd^-level comparison of the mean cluster density for all given cluster dimensions. The mean-cross p-value comparison (blue) and lower/upper confidential bounds (grey dashed line) are shown. (e) and (f) represents 600 clusters localized within the CD41/CD62p distribution respectively, displayed using the Delaunay-triangulation method (clustering dimension 390 nm). (g) shows two representative clusters from from (e) and (h) from (f).

To simplify the comparison, we determined a measure for sample similarity combining all cluster dimensions. We defined two similarity measures, *sim*_*M*_ and *sim*_*L*_. *sim*_*M*_ is the rescaled aggregated p-value with an interval of [0,1]. If *sim*_*M*_ is < 0.5, the parameters describing clusters are dissimilar (see Fig 2C zoom-in). *Sim*_*L*_ measures the dependency of the critical area (spanning the cluster dimension/sizes interval and the significance level) and lower bound of the confidence interval of the aggregated p-value and delivers a value within the interval of [0,1].

### Machine learning

In order to combine all features necessary for the similarity classification, we use a machine learning method, a MLP neural network, as a classifier of sample similarity [71, 79–81] (Fig 1B). The neural net consists of three layers: an input layer, a hidden layer and an output layer. Except for the input nodes, each neuron uses a nonlinear activation function. The network requires the preparation of input data for characterization; and it applies the previously determined statistic-based features of the dataset in the training and classification mode.

**Fig 3:**
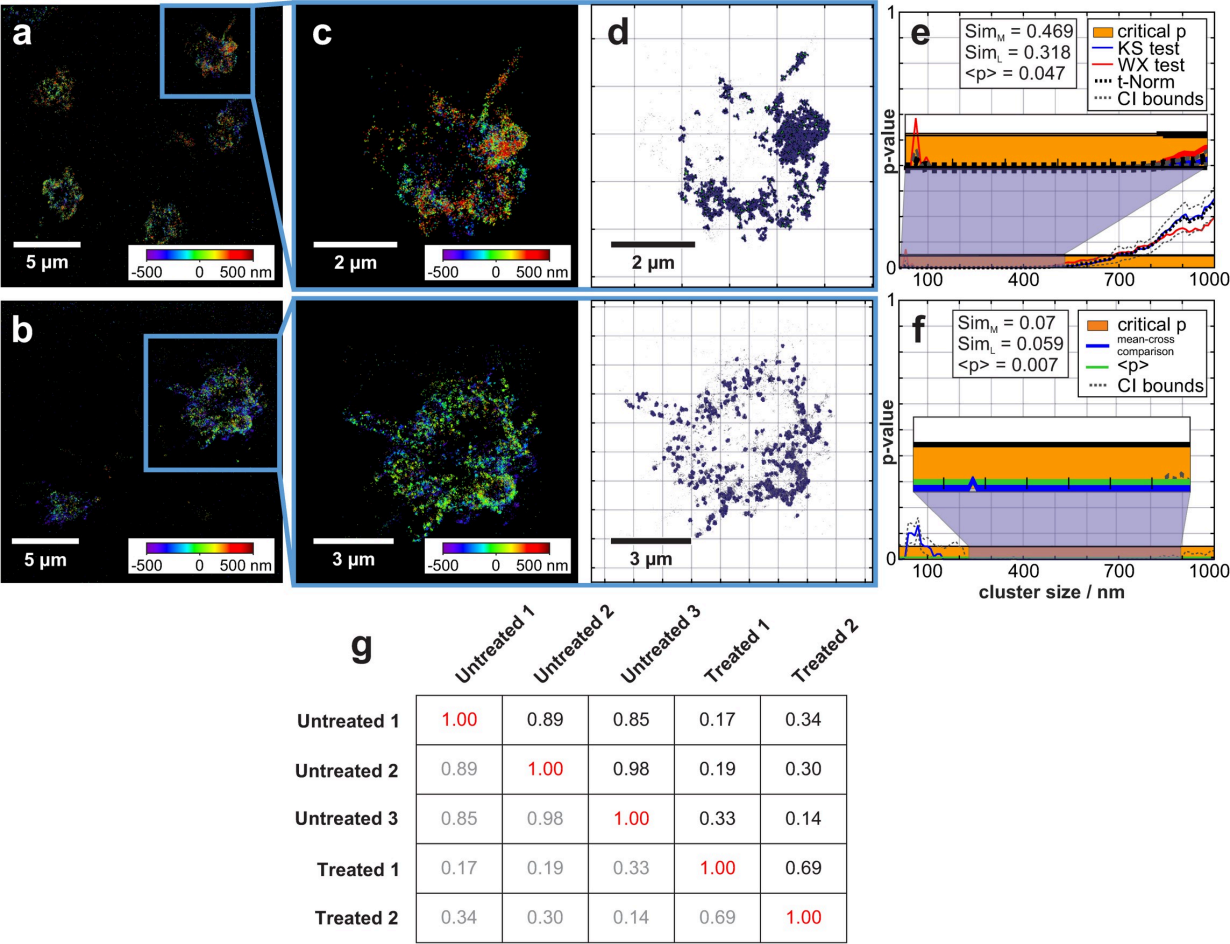
Comparison of IL-1β treated and untreated clot samples. (a) and (b) show reconstructed dSTORM images of two clots, untreated (a) and treated with IL-1β (b). In (c) zoomed images of two regions extracted from (a) and (b) are shown. In (d) two populations of clusters from both images with a cluster dimension of 145 nm are visualised. (e) shows the comparison of cluster densities for all given cluster dimensions between the two datasets (1^st^-level statistic). Blue and red lines depict the results of the KS- and WX-test, respectively [75, 88]. The aggregated p-value between KS and WX tests (black dashed line) remains below the critical p-value area (orange bar) and thus proves the dissimilarity hypothesis for most of the cluster dimensions. (f) shows the aggregated p-values of averaged density and curvature distribution, determined via mean-cross analysis (blue) and the average p-value (green, including confidential intervals: black dashed lines) (*sim*_*M*_ = 0.07 and *sim*_*L*_ = 0.06). The table in (g) shows the classification table with MLP neural network classification values for dissimilar and similar samples. The output values of the trained network can be interpreted as *a posteriori* probability of similarity hypotheses.

We trained the MLP neural network with a data set of CD41/CD62p protein distributions in an artificial clot. Approximately 262 000 training patterns were determined using this data set. The trained MLP neural network classifier was tested on ~40 different pairs of data sets, which were not included in the training set. All of the tests showed a similarity classification with a probability between 0.75 and 0.95 for a pairwise comparison of similar samples and <0.2 for a pairwise comparison of dissimilar samples (S2 Table), i.e. the parameter extraction presented in the analysis sets is a suitable base for machine learning based pattern recognition. The results on MLP neural network classification for a representative statistical comparison of sample pairs are presented in Fig 3G. The overall values of further data sets are show in the S2 Table.

### Proof-of-principle: Comparison of CD41 and CD62p clusters

In Fig 2, we show a typical reconstructed image of two areas in the central region of the clot, which visualises the distribution of CD62p (Fig 2A) and CD41 (Fig 2B) upon platelet activation (~ 100 000 single-molecule localisations each). The reconstructed images have been compared using the developed statistical tools, proving a dissimilarity of both datasets at a very detailed level.

### 1^st^-level statistics: KS/WX tests of cluster density and curvature

For each pair of data sets (or regions) hierarchical spatial clustering is performed [49–52]; i.e. we performed a 1^st^-level analysis for the relative density (Fig 2C) and curvature [82] (comparison data: S1A Fig) for all cluster dimensions of a given interval (typically from 5 nm to 1000 nm). Samples are typically equalized via the bootstrap method (100 curves for relative density and curvature of 100 re-samples all including 8000 points). Based on KS and WX tests, a direct comparison of the clusters density distribution and curvature distribution is performed (Fig 2C and S1D Fig). The determined p-values disprove the hypothesis of both samples being dissimilar. The aggregated p-value between KS-(blue) and WX-(red) tests = 0.04, and the similarity measures *sim*_*M*_ = 0.39 and *sim*_*L*_ = 0.45 indicate a difference in the cluster density distributions for all clustering dimensions between the two samples. The detailed density comparison proves that for clustering dimensions larger than 700 nm both samples have a significant similarity (Fig 2C). Further 1^st^-level analysis results on cluster curvatures (shape) [82] are presented in S1A–S1D Fig. S1C Fig represents the aggregated data of KS-/WX-test results on the characterization of cluster curvature for all clustering dimensions. The detailed curvature analysis indicates a similarity for clustering dimensions larger than 500 nm up to 1000 nm.

For the combination of the results of various tests, the calculated p-values of the individual ones are aggregated using the t-norm-AND operator (AND operation in fuzzy logic). For the similarity measure, the p-values are further transformed into *sim*_*M*_ and *sim*_*L*_ values by averaging the aggregated p-values and taking into account their confidence intervals. The process of hierarchical aggregation of individual p-values as shown in S2 Fig.

In conclusion, the results of averaging the KS-/WX-test values for cluster densities and curvatures of the bootstrapped re-samples indicate a strong dissimilarity of the samples for the maximal clustering dimension of an interval of 5 nm-1000 nm.

### 2^nd^-level statistics: Mean-cross comparison of cluster density and curvature distribution

2^nd^-level statistics were performed solely for the bootstrap derived re-samples (Fig 2D and S1E–S1I Fig). This relies on a mean-cross p-value obtained from cross-comparison tests (KS/WX) of the averaged cluster density distributions between all bootstrap resamples. Fig 2D shows the 2^nd^-level comparison of the mean cluster density for all given cluster dimensions. The mean-cross comparison for curvature distributions is presented in S1E Fig with mean-cross p-values and lower/upper confidential bounds. The mean-cross p-value (KS-/WX-test) of the density distributions equals 0.049, and *sim*_*M*_ = 0.49 and *sim*_*L*_ = 0.30 underlines the nanoscopic dissimilarity of the two samples. Similar to the results on density analysis, we determined the behaviour of the cluster curvature (S1E Fig); a mean-cross p-value for curvature of 0.23 and *sim*_*L*_ = 0.34 indicates a dissimilarity between the two samples. Comparable to the results of the 1^st^-level statistics, only for cluster dimensions larger than 700 nm the curvature as well as density distributions indicated a stronger similarity. The mean-cross p-values of the cluster curvature show a tendency towards sample similarity for cluster dimensions ~200 nm.

Additionally, the individual clusters can be visualised three-dimensionally using the Delaunay-triangulation method [83, 84] or as maximum radius spheres packed at given locations [85] (Fig 2G and 2H). Fig 2E and 2F show 600 clusters from both datasets (cluster dimension 390 nm). In Fig 2G and 2H two randomly chosen clusters have been depicted from e and f, respectively. The software feature for cluster visualisation provides detailed information on localisation of the clusters within the clot for each clustering dimension.

For large clustering dimensions, clusters cover large parts of a platelet (size 1–5 μm, see S11E–S11G Fig) and can therefore be regarded as ‘bulk signal’ by looking at micrometer-sized clusters in the nanoscopic scale regime. Hence, the results on a nanoscopic and microscopic level for dataset comparison are provided simultaneously. Further analysis (S1F Fig) displays the aggregated p-values of a mean-cross analysis for the cluster densities and curvatures (for all cluster dimensions). These aggregated p-values remain below the boundary of the critical p-value for all clustering dimensions with a dimension smaller than 700 nm. The aggregated p-values of the mean-cross analysis for the cluster densities and curvatures again indicates a dissimilarity of the samples.

### 2^nd^-level statistics: K-/H-Ripley’s functions

For further 2^nd^-level statistics we performed a Ripley’s K-/H-analysis [70, 86, 87] (S1G–S1I Fig) on the bootstrap sample data. The K-/H-Ripley’s function values are determined for each clustering dimension and are used to additionally prove the diversity of the two samples. The comparison of the values from the KS-/WX-test applied on the 3D K-/H- Ripley’s function results for each of the cluster’s dimensions (shown in S1H Fig). The results show an average p-value of 0.087, *sim*_*M*_ = 0.71 and *sim*_*L*_ = 0.14 for the K-Ripley function (left) and an average p-value of 0.086, *sim*_*M*_ = 0.71 and *sim*_*L*_ = 0.22 for the H-Ripley function (right). Additionally, the mean-cross analysis of the K-Ripley function distributions represented in S1H Fig are shown in S1I Fig. The overall Ripley analysis (*sim*_*L*_) confirms the sample dissimilarity except for a small interval of the cluster dimension (400 nm– 600 nm). For these samples (Fig 1), the comparison based on the Ripley functions confirms the analysis performed with the clustering methods.

### 1^st^-level and 2^nd^-level statistics: MLP neural network

A multilevel analysis yields a general dissimilarity between CD41 and CD62p spatial distributions within a clot for the presented data sets. Additional data sets and analysis results are shown in S3–S8 Figs. We present in detail a pairwise comparison of CD41 distributions and CD62p distributions in distinct clots. In contrast to the sample presented in Fig 2, some CD41 and CD62p distributions in clots cannot be unambiguously discriminated against by individual statistical tests. Therefore, multilevel cross-testing is explicitly required. In particular, the data presented in S7 Fig and S8 Fig show divergent results regarding cluster comparison. For these technical replicas, a significant difference between the results of the 1^st^-/2^nd^-level cluster-based analysis and the results on K-/H- Ripley’s analysis for all cluster distances is observable (S8 Fig (cluster analysis) and S8F–S8I Fig (Ripley analysis). The K-/H- Ripley’s analysis indicates a strong similarity for clusters larger than 400 nm, which is not the case for all other 1^st^-/2^nd^-level cluster-based statistics. The calculation of the mean-cross p-value for all the parameters rejects the null hypothesis of similarity, showing that both datasets are dissimilar (S8H Fig). All statistical p-values are represented in S1 Table. The data comparison confirmed a significant dissimilarity for cluster dimensions. First level analysis shows a dissimilarity (*sim*_*M*_ = 0.006 and *sim*_*L*_ = 0.5 for density and curvature comparison, respectively). A higher similarity between the curvatures occurs only for cluster < 150 nm. Second level analysis indicates a general dissimilarity (aggregated *sim*_*M*_ values are 0.6 and 0.3 for density/curvature comparison, respectively). The mean-cross comparison of all results indicates a strong dissimilarity (*sim*_*M*_ = 0.3). The Ripley’s K-/H-functions show a higher similarity *sim*_*M*_ = 0.67/0.69. The MLP neural network indicates a 0.74 probability for dissimilarity. A comparison of a clot sample (CD41 labelled) and simulated data is presented in S13 Fig. In the last analytical step the MLP neural network was applied for a comparison of the protein distributions of CD41 and CD62p in the platelet clots. The MLP neural network analysis showed that the samples were classified as similar with *a posteriori* probability of ~0.95 for a pairwise comparison of similar data sets and below 0.2 for a pairwise comparison of dissimilar ones. The *a posteriori* probabilities of the similarity hypotheses are depicted in S1 Table. The trained MLP neural network clearly discriminates between the two clustered protein populations under investigation within clots.

### Characterization of CD62p clusters after platelet activation by the cytokine IL-1β

To quantify the effect of IL-1β on platelet activation, our software toolbox has been used to analyse dSTORM data of CD62p secretion. Various cytokine-treated and untreated samples were investigated. Due to IL-1β treatment, heterogeneities in clot formation (more sparsely distributed platelets) are observable. In order to capture the best overall picture of CD62p membrane incorporation after IL-1β treatment, images with varying cell densities were compared. We observed that the numbers of clusters changes significantly depending on clustering dimension. Herein, for cluster dimensions of 80 nm, 3620 and 9106 clusters were detected in total, whereas for cluster dimensions of 145 nm, 1849 and 5205 clusters were detected in treated and untreated samples, respectively. Furthermore, data obtained from sparse and dense clot regions were analysed. For a comparison of regions with sparsely distributed platelets, the best results were obtained for segmented images. The DBSCAN-derived segmentation is crucial for 2CALM analysis, especially in cases of sparsely distributed platelets; the unspecific signal outside the regions of interest exerts an influence on direct cluster comparison (for full image comparison). A detailed description of the differences in the comparison between segmented and full images is shown in Fig 3 and S9 Fig. Fig 3A–3D depict overviews and randomly chosen regions (image segmentation) for analysis in a cytokine-treated and untreated clot, respectively. Fig 3E depicts a direct 1^st^-level comparison of the clusters density distribution (curvature distribution is shown in Supplementary S6B Fig) in the chosen regions (S9A Fig). As can be seen in Fig 3E, untreated and treated samples gain similarity for larger cluster dimensions (> 800 nm). This difference was observed reproducibly for large as well as small extracted ROIs.

The 1^st^-level analysis results of cluster density and curvature indicates that both samples are in general dissimilar, whereas for clustering dimensions larger than 800 nm, both samples show a weak similarity. Overall, the *sim*_*M*_- and *sim*_*L*_-values determined for the 1^st^-level analysis are 0.46 and 0.31, respectively. With regards to cluster density, the comparison indicates that only for cluster dimensions larger than 800 nm a similarity of these two samples can be observed. The 2^nd^-level analysis of curvature confirms the dissimilarity hypothesis (*sim*_*M*_ = 0.46 and *sim*_*L*_ = 0.45) (S9B Fig). The mean-cross comparison of the aggregated KS-/WX-test values for density and curvature also shows a dissimilarity (Fig 3F).

The comparison of the averaged cluster curvatures tends to gain similarity for clustering dimensions larger than 500 nm (S9B Fig). The mean-cross analysis of the combined statistical data on 1^st^- and 2^nd^-level cluster density and curvature comparison is shown in S9C Fig. Results from the K-/H-Ripley-analysis support the cluster comparison data S9D and S9E Fig (K-/H-Ripley’s-function (K-left, H-right) comparison for *sim*_*M*_/*sim*_*L*_ are 0.03/0.01 and 0.04/0.01, respectively). The aggregation values of these data sets show similar tendencies (S9E Fig). A comparison of CD62p protein distribution and clusters for IL-1β treated and untreated samples verifies the effect of this cytokine on platelet activation in cluster formation. For clustering ranges between ten and a few hundred nanometres, the data sets differ most; this effect has not been observed in such detail before (see S2 Table).

The MLP neural network was used for a comparison of IL-1β treated and untreated clots. The neural network clearly classifies the samples based on the CD62p protein distribution. The tests showed a classification with a probability of 0.9 for a pairwise comparison of similar data sets and below 0.2 for a pairwise comparison of dissimilar ones. The *a posteriori* probability of similarity hypotheses are depicted in S2 Table. The results of the IL-1β treated samples are remarkable: In general, a larger heterogeneity within the group of treated samples in comparison to untreated samples can be observed (similarity probability = 0.67 for MLP comparison of cytokine-treated samples; similarity probability > 0.8 for MLP neural network comparison within the group of untreated samples, Fig 3G).

## Discussion

In this study, we demonstrated that 3D LM (dSTORM) with subsequent advanced statistical analyses can be used as a tool to determine and classify differences in protein distributions between two datasets. We have successfully used 2CALM for a comparison of CD41/CD62p distributions in platelets within a clot and for determination of the effect of IL-1β-treated platelets on CD62p membrane distribution.

The highly abundant CD41 (part of fibrinogen receptor GPIIb/IIIa) primarily binds fibrinogen, which bridges the actin cytoskeleton with the extracellular matrix to provide mechanical stability. Clustering of this membrane protein is known to be required for full activation of signal transduction (together with receptor occupancy) and acts as a signalling centre resulting in formation of focal adhesions [22]. The standard activation marker CD62p stabilizes platelet aggregates, which are formed by GPIIb/IIIa-fibrinogen interaction. Clustering of this activation marker has not been analysed previously.

Within this study, we observed apparent differences in the distributions of these two membrane proteins within a clot: While CD41 is distributed over the entire cell surface (see Fig 2A), CD62p’s differing spatial arrangement can also be found concentrated on cell edges (see Fig 2B) and in the central region of platelets in the “fried-egg” morphology. This is caused by squeezing of organelles and granules during spreading [26, 32]. It is important to note that CD41 distribution at single cells’ edges within the clot are located higher in z (app. 750 nm) than those within the cell area (Fig 2A), pointing towards the presence of inactivated, discoid cells within the clot–a phenomenon that has previously been reported in literature about murine thrombus formation [89, 90]. In Fig 2B, CD62p-positive vesicles of 400 nm– 800 nm in size most likely constitute microvesicles, which are known to be rich in CD62p and have a corresponding size of 100 nm up to 1 μm [91]. CD62p clustering (as clearly demonstrated in this study) may serve the formation of microclusters in order to support cell adhesion. A comparison of the CD41 and CD62p on a nanoscopic level in a 3D clot has not been addressed previously. Few studies, alongside other findings, show changes in the distribution of either CD41 or CD62p upon activation in platelets [46, 92, 93]. In general, our nanoscopic comparative analysis of CD41/CD62p cluster distributions shows a dissimilarity between the samples. The results on the 1^st^- and the 2^nd^-level statistics are in accordance and correlate well with the obtained Ripley’s statistic. For a comprehensive quantification, the detailed comparison results on 1^st^- and 2^nd^-level statistics have been combined to teach a MLP neural network, which automatically classifies the samples. In S3–S6 Figs, we have shown the results of a comparison of two CD62p and of two CD41 labelled samples. In both cases, a high similarity of comparison levels has been determined. S8 Fig represents a comparison of different CD41 and CD62p labelled samples. In this experiment, the Ripley analysis does not match the more detailed results on cluster comparison. These results demonstrate that an analysis taking into account multiple sample-derived parameters provides a reliable input dataset for the MLP neural network.

Previous reports as well as preliminary experiments (S12 Fig) indicate that the spatial pattern of CD62p transported to the plasma membrane from the α-granules changes significantly upon IL1-β treatment [94]. We used our software platform 2CALM to compare the CD62p distribution of platelets in clots that are either untreated or treated with the inflammatory cytokine IL1-β. As shown in S9 Fig and Fig 3, results on comparison of cluster density and curvature diverges between the two compared data sets. Our results clearly show that CD62p distributes and clusters differently upon platelet activation by IL-1β treatment confirming previous reports in the nanoscale regime [92]. Herein, we observed a lower cluster number for clusters of different size (80 nm and 145 nm for example) for cytokine-treated samples.

The 2^nd^-level statistical results for the pairwise comparison of data in ROI’s yield *sim*_*M*_ values of 0.7 ± 0.2 for cluster density and 0.5 ± 0.2 for cluster curvature.

A correlation between ROI size and data set similarity can be observed. This indicates that CD62p protein clustering is affected when platelets interact with each other (compared to individual platelets in a clot) as is expected considering its role in homo- and heterotypic contact formation.

We showed that CD62p protein distribution in a clot changes upon cytokine-stimulation (Fig 3). The 1^st^- and 2^nd^-level statistical comparison, as well as the Ripley’s statistics show that densities and curvatures of the formed clusters differ significantly for all cluster dimensions. The MLP neural network classification allows for determination of the class (treated/untreated) with a 100% accuracy for all pairwise comparisons of the measured samples (S2 Table). Additionally, we showed that although the analysis indicates a general similarity of treated samples only, crowded platelets show slightly different clustering behaviour when compared to individual platelets embedded in a clot (S14 Fig).

We have been able to show that our system allows the examination of time dependent behaviour of platelets induced by external factors, by taking a time series of samples (S13 Fig). In case at a certain timestamp a dissimilarity is detected, it is possible to further analyse which cluster sizes interval shows the difference. These clusters can be filtered and further analysed regarding their dynamic behaviour in time. Cluster visualisation can be seen as performed on individual clusters and for the whole sample by creating a triangulation image.

Recently, research has been conducted on deep-learning based 3D-point clouds classification and segmentation [95]. These methods, however, require a multitude of samples for training, are time-consuming and computationally inefficient for large numbers of points in the clouds [95]. By employing a MLP neural network, we were able to combine the copious determined parameters, ultimately allowing for the classification of sample similarity. MLP neural network simplifies the comparison and extracts a combined measure, determining the probability of two data sets either being similar or dissimilar. We classified the CD62p distribution on IL-1β-treated and untreated samples using this particular machine learning approach. The high classification accuracy of the MLP neural network confirms the efficient parametrisation for the cluster-based analysis. The MLP neural network classifies crowded and individual platelets as the same class. However, the detailed 1^st^-/2^nd^-level statistical comparison precisely identifies the cluster dimensions, for which both samples show the highest similarity and dissimilarity.

Extracted spatial features based on statistical tests are directly used to create and train MLP neural networks. As the MLP neural network tests have shown, it is very robust even in case of controversial results of particular statistical tests. The training set also includes features in which the Ripley’s K-function test is false as opposed to correct classification by the first statistical level. In neural network learning, if level one retail features are consistent with level 2-features, this will increase the probability of correct sample similarity classification. The features indicate wrong estimations. If the classification acceptance threshold is exceeded (e.g. posteriori probability <60%) this will be a signal of bootstrapped re-sampling repetition with an increased number of points and possibly filtering the noise.

The presented system has following advantages and limitations: There are no restrictions in types of samples, various distributions of localisations (e.g. random, concentrated and fibrous) have been correctly classified by MLP neural network and analytical similarity measures. MLP neural network is robust for opposite level classifications and can easily be extended to multi-channel CNN. The analysis gives correct classifications in contrast to often ambiguous classifications with standard methods, e.g. Ripley’s. Our program allows short calculation time regardless of sample size and sample type (resampling of 2000–10 000 points) and the methods used are suitable for parallel computing.

The limitations of the system concern the global structure of samples. Clouds with a large number of outlying localisations (noise due to unspecific protein binding) require filtering. Single outlier points deteriorate cluster density statistics. Samples with multiple separate segments (cells or cell structures) require regionalisation, which is long-lasting. High calculation time are required for bootstrapped samples containing above 50 000 localisations.

Clustering based analyses of LM datasets opens up a unique parameter range for sample classification. We have shown that the designated parameters (like KS/WX p-values for cluster density and curvature similarity, and mean-cross values of the tests) are sufficient to correctly classify any data set of a selected population. With this, the newly developed 2CALM platform is well suited for a pairwise comparison of protein distributions in healthy, pharmaceutically treated tissues. Moreover, 2CALM is also suitable for a comparison and classification of protein distributions on any other cell type. The provided statistical tool is versatile, applicable for any pairwise LM dataset and provides an essential tool for shedding light on protein distributions, which are detectable only at a high-resolution level.

The sequential calculation of the Ripley’s K-function for an incremental radius as presented by Owen et al. gives one global characteristic of the density distribution of sample clusters. In our case, as shown by simulations and exemplary experimental data (S13 Fig) in particular for samples presenting polarised fibrous structures like actin, such a global approach can give an ambiguous or false estimation of sample similarity. The Ripley’s functions K and H in our analysis pipeline can be seen as auxiliary additional features, sharpening estimates for the probability of sample similarity using machine-learning methods.

Analysis systems such as SR-Tesseler [18] or ClusterVisu [19] are based on Voronoi tessellation show great potential for either visualization and rendering of localisation events density distribution in a sample or colocalization between two-color localisation events. It has been demonstrated for 2D datasets with relatively low numbers of localisations. The Voronoi tessellation cell represent the ‘area of influence’ for localisation inside a single cluster. This corresponds to building of density distribution from single-point clusters using hierarchical clustering with small size radius, e.g. 1 nm or 2 nm with our software. The single point cluster density determined by our software in nearest proximity to SR-Tesseler, can be regarded as invers area/volume of Voronoi cell. However, in this case the sample density distribution becomes very sensitive to the number of localizations. Additionally, the computation time is high for large samples.

The method proposed by Burguet et al. [96] is based on comparison of the intensities of points at local spatial positions within the samples [97]. The method requires normalization and correction of spatial data. That way all localisation positions in the samples are expressed in the same 2D / 3D coordinate system. Within this normalized coordinate system, the number of points and their positions may vary depending on the spatial structure of the sample, which creates variations in the analysis. The proposed solution is based on the local intensity estimator, which than creates an intensity map for each sample and tests for local intensity differences. Secondly, the method has up to now never been used for LM applications. In contrast to the algorithm developed by Burguet, our software does not require synchronization of the coordinate system and the size of the observed region (i.e. ROI) or possible normalization of both samples.

The interpretation of the methods typically requires a multimodal analysis. Nowadays, correlative approaches combining multiple methods are used. The main goal of this work is to establish a platform which is based on multiple parameters yielding one global answer on two-sample similarity (regardless of the rotation or shifting of the localizations cloud). The software we developed however allows accessing the comparison data on each analysis detail-level and extract the information on similarity for each cluster dimension. This information can as complement information’s derived from other nanoscopic imaging method like Atomic Force Microscopy (AFM) or Electron Microscopy (EM).

## Methods

### Equalization and bootstrap resampling

We use inferential statistics to examine the relationships between the features of two samples based on a series of smaller samples in order to generalize how those features will relate to the larger sample [67, 36, 68, 69, 98–103]. For analysis, we chose representative subsets of two samples, which will be referred to as the bootstrap-resamples. The bootstrap procedure involves choosing random samples (with replacement) from a large dataset and analysing each bootstrap sample in a similar way. Sampling with replacement means that each point is selected randomly from the original dataset. Thus, a particular point from the original dataset may appear multiple times in a given bootstrap sample. The number of total points included in a bootstrap sample (including data from both compared samples) is equal. Let *N*_*1*_, *N*_*2*_ be the number of points in both samples, respectively. We perform *M* times random resampling of each sample with the same number of points *N*<*min*(*N*_1_,*N*_2_). Default values for *M* = 100 and *N* is ~2000–10 000 points. These bootstrap-samples (*bs*^1^(*k*),*bs*^2^(*k*))(*k* = 1,…,*M*) are pairwise stored for further analysis.

### Spatial clustering via hierarchical clustering

Hierarchical clustering (also called Hierarchical Cluster Analysis (HCA)) is a common algorithm, which creates a hierarchy of clusters [49–52]. The agglomerative approach at the beginning declares each point as an individual cluster. Thus, pairs of clusters merge as one-element cluster moves up the hierarchy. Hierarchical clustering creates a cluster tree or dendrogram. This tree is a multi-level hierarchy with clusters of one level being joined to clusters in the next level. Grouping of the clusters into a tree connects pairs of clusters, which are close together by using a linkage function. The linkage function uses the distance information between points to determine the proximity of clusters relative to each other. Next, newly created clusters are grouped into larger clusters until a hierarchical tree is created. We apply a complete-linkage clustering function, which uses the maximum of the pairwise distances between points for clustering [51, 52].

For data partitioning, we cut the hierarchical tree into clusters with a given maximal cluster dimension/cluster size (maximal Euclidean distance between points inside a cluster); e.g. at the level of *d = dim(i)*, where *dim(i)* is a vector of cluster dimensions, by pruning off branches from the bottom of the hierarchical tree, and assigning all the clusters below each cut to a single cluster.

The use of complete-linkage hierarchical clustering guarantees that agglomerated clusters have a dimension (*size*^*Cluster*^) no greater than given *dim(i)*. In the hierarchical clustering process, cluster sizes *dim(i)* are ordered sequentially from *size*_*min*_ to *size*_*max*_ with a constant Δ step. In order to find features that allow comparison between two samples, both samples are clustered sequentially with a given maximum cluster size *d* = dim(*i*), where *dim*(*i*) = *size*_*min*_+*i*∙Δ, and *i* = 1,…,*L* and *L*∙Δ = *size*_*max*_.

For linkage, the *size*_*max*_ for both clustered samples is assumed to be ~ 40% to 50% of the minimum dimension of both samples. The dimension of the sample can be defined as the minimum length of the 3D box, which includes all points of the sample. We set the *size*_*min*_ between 5 nm-10 nm and the step Δ to be 10 nm as default values.

### Cluster parameter extraction

The platform allows extraction of **6 sample-independent parameters:**

- Cluster density
- Cluster curvature
- Average cluster density
- Average cluster curvature
- Ripley’s K function
- Ripley’s H function

Let *Cl(d)* be the set of *N(d)* clusters for one of the samples (1 or 2) with the cluster maximum size *d = dim(i)* (for sake of clarity, we will omit the *i* index from now on).

### Cluster density

For each cluster *c*_*k*_ (where *k* = 1…*N*(*d*)) from the set Cl(d) one can calculate its density ${dens}_{k}=n_{k}\frac{1}{V_{k}}$ and relative density as *density*_*k*_ = *dens*_*k*_/*density*(*sample*), where *n*_*k*_ is the number of points in k^th^-cluster from the set *Cl(d)*, *V*_*k*_ is its volume and the $density(sample)=\frac{N}{V}$ is the density of the whole sample.

The density of the cluster slightly depends on the method of determining its volume. The platform allows one to use three different methods to determine the cluster volume:

1. Sum of the sphere volumes with the sphere centre in each point of the cluster and the radius equal to the half-distance to the nearest neighbour (called bullet-density, S10E Fig) [84].
2. Volume of convex hull spread upon cluster points (called hull-density, S10F Fig).
3. Volume estimated as a volume of a rectangular box including cluster points (called box-density, S10G Fig).

As default, we use the bullet-density that best reflects the shape of the 3D cluster.

### Cluster curvature

The value of the curvature reflects the concave-convex degree of the cluster surface. We use the method presented by He et al and Williams and Shah [82, 104] to estimate the curvature of the cluster by analysing the covariance between all cluster points. For a 3D cluster, we determine the covariance matrix of each point in a cluster based on a centroid calculation.

Let *pos*_*k*_ *= (x*,*y*,*z)*_*k*_ be a matrix of the 3D point locations in a cluster *c*_*k*_ from the set *Cl(d)* and *p*_*k*_ be the centroid of these points. The *pos*_*k*_ is a *c*_*k*_ *x* 3 matrix. The 3D covariance matrix *COV*_*k*_ = ∑_*j*_(*pos*_*k*_(*j*)−*p*_*k*_)∙(*pos*_*k*_(*j*)−*p*_*k*_)^*T*^ where *j* = 1,…,*n*_*k*_ is a semi-positive definite three-order symmetric matrix. Next, the three eigenvalues of the *COV*_*k*_ matrix λ_1_, λ_2_, λ_3_ and its corresponding unit eigenvectors *ev*_*1*_, *ev*_*3*_, *ev*_*3*_ are calculated. Let λ_1_ ≤ λ_2_ ≤ λ_3_. Eigenvalue λ_1_ describes the change of the value of the surface along the normal direction. The surface variation can be expressed as 𝜏_*k*_ = λ_1_/ (λ_1_+ λ_2_+ λ_3_). The curvature *curv*_*k*_ of the cluster *c*_*k*_ can be approximated as a surface variation 𝜏_*k*_ [104].

Both features, densities and curvatures of clusters, can be used for first level comparison or detailed level sample comparison. The relative density and curvature of clusters with a given dimension *d* can be considered as empirical distributions with an unknown probability density function (pdf). The empirical distributions of both samples can have a different number of elements (different number of clusters).

Based on these detailed distributions, we can also specify the global features of the clusters used later in the second level analysis. For each cluster size *d* = *dim*(*i*), the two additional sample features can be determined:

**Average density of clusters:** *av*_*density*(*d*) = *mean*(*density*_*k*_|*k* = 1,…,*N*(*d*))

**Average curvature of clusters:** *av*_*curv*(*d*) = *mean*(*curv*_*k*_|*k* = 1,…,*N*(*d*))

### 3D Ripley’s K-function and Ripley’s H-function

Sequential clustering is simultaneously used to calculate the value of the Ripley’s K-function for each sample [70, 86, 87]. Ripley's K-function is an intuitive approach for detection of deviations of general assumptions of point distributions within cluster samples. The analysis has a non-parametric character and is therefore the first step in the characterization of spatial point patterns. The K-function can be calculated for each size *d = dim(i)* of clusters between given *size*_*min*_ and *size*_*max*_.

Let *Dist(l*,*k)* denote the Euclidean 3D-distance matrix between each pair of points in the sample. An extension of the K-function from 2D to 3D is obtained by assuming 3-dimensional distance measures.

Thus, the **K**-function for a given distance *d = dim(i)* is determined as

$$K(d)=\frac{V}{N^{2}}\cdot \left(\sum_{k=1}^{n}\sum_{l\neq k}e_{k}(d)\cdot I[Dist(k,l)\leq d]\right)$$ (1)

where *V* is the volume of the sample, *N* is the number of sample points, *e*_*k*_(*d*) is the edge correction term for a sphere of radius *d* with *k* point as its center and *I*[∙] is the indicator function [70]. The expected value of the complete spatial randomness (*CSR*) is *E*[***K***(*d*)] = 4*πd*^3^/3 as well as the function [86, 87]:

$$H(d)=\sqrt[{3}]{3K(d)/4\pi }\;d$$ (2)

Finally, for each sample, six features can be determined. The sample is sequentially divided into clusters with dimensions *dim*(*i*) = *size*_*min*_+*i*∙Δ. For each size *d* = *dim*(*i*) the sample is represented by:

- Vector ***density***(*d*) of the relative density of clusters for size *d*
- *Curvature vector* ***curv***(*d*) of clusters for size *d*
- Value ***av_density***(*d*) of average density of clusters
- Value ***av_curv***(*d*) of average curvature of clusters
- Value ***K***(*d*) of the Ripley’s function
- Value ***H***(*d*) of the Ripley’s function

Using H-Ripley as the crucial dimension, representative for the largest difference (clustering caused) between the sample and complete spatial randomness can be determined.

### Sample comparison based on extracted parameters

Regardless of whether comparisons are carried out on full samples or selected ROIs, we derive the above-described features for each given *d* = *dim*(*i*). For every *d*, samples are compared using the results of the Ripley functions ***K***_***1***_, ***K***_***2***_ and ***H***_***1***_, ***H***_***2***_, density and curvature distributions ***density***_***1***_**, *density***_***2***_**, *curv***_***1***_**, *curv***_***2***_ clusters of samples 1 and 2, respectively. Cluster density distribution ***density*** and the distribution of cluster curvature ***curv*** are two random variables with an unknown probability density function.

### 1st-level analysis

For two cluster density distributions ***density***_***1***_ *and* ***density***_***2***_ originating from sample 1 and 2 the null hypothesis states that both distribution ***density***_***1***_ *and* ***density***_***2***_ belong to the same original distribution. To resolve the validity of this hypothesis at a given significance level α, we use two-sample Kolmogorov–Smirnov test [72–74].

Kolmogorov-Smirnov (KS) test compares empirical cumulative distribution functions based on their absolute differences. The null hypothesis is rejected at a significance level α in case the difference exceeds the critical value. The KS-test returns an asymptotic p-value as a scalar value in the range [0,1]. The p-value is the probability of observing a test statistic more extreme than the observed value under an assumption of a true null hypothesis. The asymptotic p-value becomes very accurate for large sample sizes and is reasonably accurate for sample sizes *N*_*1*_ and *N*_*2*_, such as *(N*_*1*_ *N*_*2*_*) / (N*_*1*_ *+ N*_*2*_*) ≥ 4*. Therefore, one can accept the p-value *pv(d) = testKS(****density***_***1***_*(d)*, ***density***_***2***_*(d))* for each size *d* as one comparative variable.

The second null hypothesis assumes that both distributions ***density***_***1***_ and ***density***_***2***_ are taken from continuous distributions with equal medians. To resolve the validity of this hypothesis at a given significance level 𝛼, we can use the Wilcoxon rank-sum test (WX, Wilcoxon–Mann–Whitney test) [75, 76]. This non-parametric test is used to determine if two independent samples were selected from populations with equal distributions. The test assumes that the two samples are independent. A two-sided WX-test returns the p-value as a positive scalar in the range [0,1]. Data ***density***_***1***_ and ***density***_***2***_ can have different lengths.

Therefore, in addition to the p-value of the KS-Test, one can calculate the p- value *pw(d) = ranksum(****density***_***1***_*(d)*, ***density***_***2***_*(d))* for each size *d* of the WX-test as the second comparative variable.

After calculating the p-values for both tests, one needs to aggregate both variables *pv* and *pw* for each size *d*. We apply the weighted t-norm-AND operator, which is often used in AND-aggregation in fuzzy logic [77, 78]. The weighted t-norm-AND operator calculates aggregated p-values as $pd=pv\;⋀\;pw=1-\sqrt{w{\left(1-pv\right)}^{2}+(1-w){\left(1-pw\right)}^{2}}$, where parameter *w* is a weight between 0 and 1 and allows to assign more significance to one of the tests. Default *w* is ~0.5–0.6.

The same method is applied to the clusters curvature distributions ***curv***_**1**_ and ***curv***_**2**_ with the given dimension *d*. As result, we obtain curves representing p-values ***pv*,*pw*,*pd*** for relative density and ***pcv*,*pcw*,*pc*** for curvature of clusters depending on the cluster size *d*. Additionally, the distribution-free multivariate KS-test allows for a comparison of the pair of distribution (***density***_***1***_, ***curv***_***1***_) with the pair of distribution (***density***_***2***_, ***curv***_***2***_) [72].

### 2nd-level analysis

The bootstrap technique provides M pairs of bootstrap samples (*bs*^1^(*k*),*bs*^2^(*k*)|*k* = 1,…,*M*). For each resampled pair, features are calculated based on the sequential clustering of both *bs* for each cluster size *d* = dim(*i*),*i* = 1…*L*. For extracted features of each pair of *bs*, the above-described first level statistic tests are performed and the results are stored in a *L*×*M* matrix. The rows of the matrix are adequate to the cluster sizes and the columns represent the individual bootstrap samples. The values of the Ripley’s K- and Ripley’s H-functions of the bootstrap results are assigned to the matrices ***KR*** and **HR.** The p-values of the KS-test and the p-values of WX-test for relative densities and curvature distribution in clusters are included in the matrices ***KD*** and ***KC***, ***WD*** and ***WC***, respectively. Additionally, the bootstrapping method stores the average values of relative clusters densities *av*_*density* and of the clusters curvatures *av_curv* into matrices ***DM*** and ***CM***, for each dimension *d* of both samples.

Next, bootstrap confidence intervals are calculated. Having multiple realizations of random variables of density distribution and curvature distribution of clusters enables the estimation of confidence intervals for p-values generated by the KS- as well as the WX-test. Alternatively, for estimation of the standard parametric confidence intervals (of known distributions) we use the semiparametric or nonparametric methods with bootstrap estimates. Hence, the bootstrapping technique estimates the standard error deviation, which is used in a normal approximation of confidence intervals for significance level *α* (inaccurate estimation). For α = 0.05 the normal confidence interval is approximated by *pd ± 1*.96 * std(pd). Alternatively, nonparametric *bootstrap percentile confidence intervals (BPCI)* can be calculated to infer the observed significance level of the variable. The bootstrap distributions of the p-values for each cluster size can be sorted, and α and (1 –α) percentiles of the sorted empirical distribution form the limits for the BPCI [98, 99, 102].

The comparative analysis of both samples is carried out on two levels: detailed (first level statistic) and global (second level statistic). At the detailed level, we use in the ***KD***, ***KC***, ***WD*** and ***WC*** matrices stored p-values of the KS-test and p-values of the Wilcoxon rank-sum test for comparison. In cases when *L* = 1 (whole sample), it is not possible to determine confidence intervals and the analysis is based only on the *pd* and *pc* curves and their aggregation with the t-norm AND-operator.

Having many realizations of random variables of densities and curvatures (*L* > 1), it is possible to carry out **cross tests** between the realizations as well as between their average values and Ripley functions. In addition to the previously described tests, we can directly calculate p-values (empirical p-values) between average values of the chosen variable of resamples from the first sample with all values of this variable in resamples from the second sample.

For the **global level** (second level statistic) comparison, we use additional matrices ***KR*, *HR*** (contain the values of Ripley’s K and Ripley’s H functions) and ***DM*, *CM*** of both series of resamples. The ***DM*** arrays (***DM***_**1**_ and ***DM***_**2**_ –matrices of resampling series from sample 1 and sample 2, respectively) contain the average values of the relative cluster densities within a given dimension (rows) for each bootstrap sample (column). The ***CM*** (***CM***_**1**_ and ***CM***_**2**_) matrices contain the average values of the cluster curvature with the given dimension (rows) for each resample (columns).

In order to compare the samples at this global level, we test two null hypotheses:

- **Mean-Cross-Hypothesis:** The average value of the mean cluster densities of all resample series determined from one original sample (*mean(****DM***_***1***_*)*) and all the resample distribution of mean densities determined from the second original sample (*mean(****DM***_***2***_*)*) originate from the same continuous distribution.
- **Cross-Hypothesis**: Randomly k-times chosen density distributions from one series of resamples (i^th^-column of matrix ***DM***_***1***_) and all the mean density distributions of the second resample series (***DM***_***2***_) originate from the same continuous distribution.

A similar hypothesis can be made for matrices ***CM***_**1**_ and ***CM***_**2**_ containing average values of cluster curvatures. The same hypotheses can be formulated and tested for Ripley’s function results (***KR*** and ***HR*** matrices) for both samples. For both hypotheses, we calculated the empirical p-value with methods previously described.

### Measure of similarity between samples

We determine one measure for sample similarity [105] using calculated p-value curves (for each cluster dimension). In cases of an unknown confidence interval (L = 1), we can only use the simplest method for calculation of a similarity factor between compared samples.

We use the average of the aggregated p-value (*mean*(***p***)) in significance level 𝛼 and transform, it into a similarity measure as follows:

$${sim}_{M}=\left\{ \begin{array}{l} \;mean(p)/2\alpha ,\;mean(p)<\alpha \\ 1-\alpha /(2\cdot mean(p)),\;otherwise \end{array}\right.$$ (3)

Based on confidence interval estimates, we can define an alternative measure of similarity: comparison of the intersection between the area below the critical boundary 𝛼 and the lower confidence interval boundary of aggregated p-value curve.

Let *d*_*interval*_ *=* [*size*_*min*,_ size_max_] be the full length of the cluster sizes interval. Thus, *cr =* 𝛼 * d_*interval*_ spans the critical area of the significant level 𝛼. The *intersec(****p****)* is the intersection of the critical area *cr* and the area above the lower bound of the confidence interval of ***p***. The measure for the similarity is defined as:

$${sim}_{L}=1-\frac{intersec(p)}{cr}$$ (4)

The average value of the aggregated p-value and confidence intervals of p-values for each size of cluster (matrix rows) are used to calculate ***sim***_***M***_ and ***sim***_***L***_ values. For a ***sim***_***M***_ and ***sim***_***L***_ value between 0.45 and 0.55, the similarity of the samples is marginal.

### Machine learning

We apply a machine learning method, namely MLP neural network as a classifier of similarity of samples [71, 79, 80] (S10H Fig). We train the MLP neural network with 11 input neurons and 20 neurons in a hidden layer with a hyperbolic tangent as activation function and two neuronal output nodes with a softmax activation function (S10H Fig). As a network training performance function, we use cross entropy measures [71]. This makes it possible to interpret the values of both outputs as a posteriori probability of samples similarity. Let us assume that we have carried out a Ripley functions and bootstrapping-based analysis of two samples and extracted features and tests on both analysis levels. We transform the results into a set of L * M vectors, where *L* is the number of analysed sizes of clusters in *dim(i)* and *M* the number of resampling processes. Each vector represents the result of tests and forms the input and output pattern of the MLP neural network.

Vector ***Input*** *= (d*,*p1*,*…*,*p10)* and ***Output*** = [0,1] (similar) or = [0,1] (dissimilar) where ***d*** is the cluster size, ***p***_***1***_ ***= pv(d)*** is the p-value derived from the KS-test for relative cluster densities, ***p***_***2***_ ***= pw(d)*** is the p-value derived from the WX-test for the relative cluster densities, ***p***_***3***_ ***= pcv(d)*** is the p-value derived from the KS-test for the curvatures of clusters, ***p***_***4***_ ***= pcw(d)*** is the p-value derived from the WX-test for the curvatures of clusters, ***p***_***5***_ ***= mean_cross***_***1***_ is the p-value derived from the cross-comparison of the average cluster densities between sample 1 and sample 2, ***p***_***6***_ ***= mean_cross***_***2***_ is the p-value derived from the cross comparison of average cluster densities between sample 2 and sample 1, ***p***_***7***_ ***= c_mean_cross***_***1***_ is the p-value derived from the cross comparison of the average curvature of clusters between sample 1 and sample 2, ***p***_***8***_ ***= c_mean_cross***_***2***_ is the p-value derived from the cross comparison of the average curvature of clusters between sample 2 and sample 1, ***p***_***9***_ ***= R_mean_cross***_***1***_ is the p-value derived from the cross comparison via the Ripley K-function of sample 1 with sample 2, and ***p***_***10***_ ***= R_mean_cross***_***2***_ is the p-value derived from the cross-comparison of Ripley K-function of sample 2 with sample 1. Output value is equal to [0,1] if the parameters describing the samples are similar or is [0,1] otherwise. For extraction of the proper network training set, K-Ripley and bootstrap tests of both sample groups are required. To get a full training set, we need to know a priori if the samples are similar or not.

### Cluster visualisation

S11A and S11B Fig are showing 3D representations of platelet clusters. To obtain a graphic representation of 3D objects, the Delaunay triangulation method was used. The Delaunay triangulation allows one to visualise the selected individual clusters in two ways: as shown in S11C and S11D Fig with a set of spheres with the centre in each point of the cluster and the radius equal to the half-distance to the nearest neighbour. As the default value for the cluster dimension (size), the dimension at maximum of the H-Ripley function is chosen. The H-Ripley functions are used to detect the presence of clustering in the data [106, 107]. Since the H-Ripley function is often used to recognize clustering, a positive value of ***H*(*d*)** indicates clustering over that spatial range whereas a negative value indicates dispersion. The value of ***d*** that maximizes ***H*(*d*)** indicates the radius of maximal aggregation (i.e. the size of clusters which contains the most points per volume). Therefore, ***d*** can be chosen as a neighbourhood radius *ρ* in the DBSCAN clustering method. It is used to extract the main (primary) clusters from the samples. The platform enables display of these primary clusters in 3D (S11E Fig). It is also possible to determine the empirical probability distribution of densities (S11H Fig) and curvature (S11I Fig) of primary clusters for both samples and their comparison using the KS-test.

The comparative analysis was performed on a standard PC with CPU i7 Intel, 1.9 GHz, 8 logical processors and 16 GB of RAM. Total analysis time for one pair of resamples and for all cluster dimensions is dependent on the number of points in a resample: e.g. for 1000 points: 0.86 s, 5000 points: 6.61s and for 10000 points: 18.11s.

### Statistics

Statistical analyses were performed using SPSS. All statistical tests are described in the Fig legends. Statistical significance value: p<0.05.

## Supporting information

S1 Methods Supporting methods. (PDF)

S1 Fig Detailed statistical results on the comparison of CD41 and CD62p clusters. This figure shows the additional results regarding the statistical comparison for the two samples presented in **Fig 2** (sample 1 has > 85 000 points and sample 2 has > 93 000 points). (**a**) shows the direct comparison of the full samples (all localisations) via a KS-/WX-test of the cluster densities (top) and curvatures (bottom). (**b**) shows the aggregated KS-/WX-test p-values of curvature calculated with t-norm-AND operator of the results represented in (**a**). (**c**) displays the values of the KS-/WX-test and their aggregation via t-norm-AND operator determined from the cluster curvature distributions received from the bootstrap resampling (comparable to **Fig 2C**). (**d**) shows the bootstrapping results for cluster density and curvature (log, and normal scale). Herein, 100 resamples (all including 8 000 points) were plotted. Using the bootstrap method allows us to gain multiple testings required for the **second level analysis**. (**e**) presents a part of the **2**^**nd**^**-level analysis** and shows results on mean–cross comparison of the density and curvature distributions of resampled image data, respectively. (**f**) presents the aggregated p-value for curvature and density comparison data at both analysis levels. (**g**) displays the results of the K-/H-Ripley functions for all bootstrap samples. (**h**) shows the KS-/WX-test results of the K-/H-Ripley data derived from both samples. On the left, the comparison of the K-functions values and on the right the comparison of the H-functions are presented. (**i)** shows the aggregated p-value for comparisons of K-/H-Ripley data.
(TIF)

S2 Fig Workflow and parameters used for the comparative statistical analysis of the platform 2CALM. The figure depicts the flow diagram of the comparative analysis, including aggregation of parameters. In the first step, the sample data (full or bootstrap-resampled data of both samples) are clustered hierarchically for cluster dimensions (size) ranging from 10 nm– 1 000 nm. In the **first level analysis** of the pairwise resampled data, the Kolmogorov-Smirnov (KS)-/Wilcoxon (WX)-tests (relative density distribution/ curvature distribution) for each cluster dimension are performed. The KS- and WX-test results for comparison of cluster densities and curvatures of both (pairwise) samples are further aggregated using the t-norm-AND operator for all cluster dimensions. The results of p-value aggregation for density and curvature are once more aggregated, giving the average result of the comparison on the first statistical level. Next, the **second statistical level** analysis is performed. Here, the averaged density and curvature for each cluster dimension and resample are used to perform the mean-cross analysis between them. The mean-cross comparison uses KS-/WX-tests for a comparison between distributions of mean clusters density of all resamples from sample 1 and all individual density distributions of resamples from sample 2. Hence, an aggregation (t-norm AND operator) of the mean-cross KS/Wilcoxon-test results (p-values) for density and curvature for all cluster dimension gives a global p-value of similarity hypothesis. In parallel, in **the second level analysis** the K-/H-Ripley functions for all cluster dimensions are calculated. Hence, the mean-cross comparison on the K-/H-Ripley functions values, (same as for mean density/curvature distributions) is performed. The KS-/WX-Test results on comparison of the K-/H-Ripley function values are combined with t-norm AND, forming an additional set of parameters.
(TIF)

S3 Fig Detailed statistical comparison of CD62p cluster distributions within two clots. CD62p was stained with Alexa647-labelled antibody. Both reconstructed images are depicted in (**a**) and (**b**) (samples include > 93 000 and >120 000 points, respectively). (**c**) shows an image of selected clusters (600 clusters per image) with a cluster dimension of 180 nm. For cluster representation, we used the Delaunay-triangulation method. (**d**) depicts two randomly chosen clusters.
(TIF)

S4 Fig Detailed statistical comparison of CD62p cluster distributions within two clots. **First level analysis** is depicted in (**a-c**). (**a**) shows the detailed analysis of the full samples. Density and curvature distributions of all clusters for each cluster dimension were compared at one time using a KS-/WX-Test. The results indicate a similarity of clusters for all cluster dimensions. As an indicator, an aggregated p-value is determined using the weighting t-Norm-AND. The p-value (black dashed line) remains above the critical p-value area (orange bar) proving the similarity hypothesis for all cluster dimensions. The corresponding similarities are ***sim***_***M***_ = 0.87/0.82 for density and curvature, respectively. (**b**) depicts the aggregated p-value calculated with the weighting t-Norm-AND (blue) and confidence interval (black dashed line). A value of ***sim***_***M***_ = 0.82 is determined. The full-sample analysis does not give the full parameter range of the analysis, just a brief overview on cluster density and curvature similarity. (**d**) depicts 100 curves–representing relative density of 100-resamples each including 2000 points (green)–used for the bootstrap based comparison of the cluster/curvature density distribution for both samples. In black, the averaged values for all dimensions are represented. The detailed analysis for the bootstrap data shown in (**d**) was performed similarly to the analysis in (**a**). (**c**) shows the KS-/WX-test compared data regarding cluster density and curvature for all given cluster dimensions. As for the detailed analysis (**d**), the KS/WX-tests (blue/red) as well as the t-Norm-AND aggregated p-values are plotted (black). Additionally, the boundaries of the confidence interval are displayed (magenta). The comparative analysis of the bootstrap derived data shows a strong similarity between the samples (***sim***_***M***_ = 0.93 for cluster density, ***sim***_***M***_ = 0.95 for curvature). (**e-i**) depict the **second level analysis**. (**e**) shows the generalized density/curvature p-values determined via a mean-cross comparison. Herein, p-values of the mean-cross comparison (blue) and lower/upper confidential boundaries (black dashed line) are shown. In general, a significance similarity between the samples is visible (***sim***_***M***_ = 0.92 for mean cluster density and ***sim***_***M***_ = 0.94 for curvature distributions). (**h**) shows the aggregated p-values for cluster curvature and density distributions (blue). The aggregated p-values show high similarities ***sim***_***L***_ = 1. (**f**) depicts the calculated Ripley’s–K/-H function values for the bootstrapped data (green) for all cluster dimensions. Using H-Ripley´, the crucial dimension (representative for the largest difference (clustering-caused) between the sample and the Poisson distribution) can be determined (for the two images, the largest difference occurs for clusters sizes of 220 nm). (**g**) depicts the comparison of the K-/H-Ripley function values at a generalized level (second level statistics). The p-values of the mean-cross comparison (blue) and lower/upper confidential bounds (black dashed line) are shown. The cross-comparison of the statistical cluster-data analysis also indicates similarity of the samples. (**i**) shows the result of a pairwise KS-test on the distribution of average cluster density and distribution of average cluster curvature for both samples. We observe only a slight dissimilarity for cluster dimensions between 200 nm– 400 nm and 600 nm– 900 nm. This result confirms the similarity of both samples (***sim***_***M***_ = 0.58).
(TIF)

S5 Fig Detailed statistical comparison of CD41 cluster distributions within two clots. In both samples, CD41 was stained with an Alexa488-labelled antibody. Both reconstructed images (include >158 000 and >142 000 points, respectively) are depicted in (a) and (b). (c) shows images of the clusters with the cluster dimension of 245 nm (600 clusters in each image). For cluster representation, we used the Delaunay-triangulation method. (d) depicts two randomly chosen clusters.
(TIF)

S6 Fig Detailed statistical comparison of CD41 cluster distributions within two clots. First level analysis is displayed in the images (a-c). (**a**) shows the detailed analysis of the full samples (both samples reduced to 60 000 points) density (top) and curvature (bottom). In this case, density and curvature distributions of clusters for each cluster dimensions were compared at one time using the KS-/WX-test (blue/red). The results indicate a similarity of samples for all cluster dimensions. As an indicator, an aggregated p-value is determined using the weighting t-Norm-AND. The p-value (black dashed line) remains mostly above the critical p-value area (orange bar) proving the similarity hypothesis. The corresponding similarities are ***sim***_***M***_ = 0.57/0.91 for the density and curvature distributions respectively. Remarkably, the density distribution shows a lower similarity compared to the curvature. A closer look at the comparison of the density distributions shows, that for small cluster dimensions between 10 nm and 150 nm as well as for large cluster dimensions ranging from approx. 1 400 nm– 1 800 nm, the distributions are within the critical p-value area. (**b**) depicts the aggregated p-value (density and curvature) calculated with the weighting t-Norm-AND (blue) and the confidence interval (black dashed line). A ***sim***_***M***_ = 0.71 is determined. For such large samples, a faster bootstrap-resampling is used. (**d**) depicts 100 curves–representing relative density of 100-resamples each including 2 000 points (green)–used for the bootstrap based comparison of the cluster/curvature density distribution for both samples. In black, the averaged curve is represented. The detailed analysis (first level statistics) of the bootstrapped samples (**d**) was performed using similar statistics as used in (**e**). (**c**) shows the detailed (first level statistic) comparison of the distributions of cluster density (top) and curvature (bottom) for all given cluster dimensions (sizes). For this analysis, the KS/WX- tests (blue/red) as well as the t-Norm-AND values are plotted (black). Additionally, the boundaries of the confidence interval are displayed (magenta). The bootstrap derived data shows a strong similarity between the samples, ***sim***_***M***_ = 0.91 and 0.95 for cluster density and curvature distributions was determined. (**e-i**) depict the **second level analysis**. (**e**) shows the p-values determined by the mean-cross analysis of the average density (left)/curvature (right) values determined via bootstrapping. Herein p-values of the mean-cross comparison (blue) and lower/upper confidential boundaries (black dashed line) are shown. In general, as expected a huge similarity between the samples is visible, ***sim***_***M***_ = 0.92 for the mean cluster density distribution and 0.97 for the mean curvature distribution was determined. (**h**) shows the aggregated p-values for the cluster mean cross comparison of curvature and the mean cross of density distributions (blue). The aggregated p-values show a high similarities; ***sim***_***M***_ = 0.91 and ***sim***_***L***_ = 1. (**f**) depicts the calculated K/-H-Ripley’s-function values for the bootstrapped data (green) for all cluster dimensions. (**g**) depicts the comparison of the K-/H-Ripley function values (K-left, H-right). The p-values of the cross comparison (red), mean-cross comparison (blue) and lower/upper confidential bounds (black dashed line) are shown. This cross-comparison of the cluster-data also indicates a similarity of the samples for Ripley’s function analysis. The aggregated p-value of the results of the K-/H-Ripley functions comparison (**i**), indicates a slight dissimilarity of the results for cluster dimensions (sizes) between 200 nm– 600 nm.
(TIF)

S7 Fig Comparison of CD41 and CD62p cluster distributions within a clot. In one clot sample, CD41 was marked with an Alexa488-labelled antibody; in the other sample, CD62p was marked with an Alexa647-labelled antibody. We used our software platform to compare the two distributions of CD41 and CD62p molecules determined from the centres of two biological replicas of clots. Both reconstructed images are depicted in (**a**) and (**b**) (both reconstructed from approx. 300 000 localisation events). (**c**) shows the image of clusters with the cluster dimension of 235 nm (600 clusters in each image). For cluster representation, the Delaunay-triangulation method is used. (**d**) depicts two randomly chosen clusters.
(TIF)

S8 Fig Comparison of CD41 and CD62p cluster distributions within a clot. First level analysis is displayed in the images (a-c). (**a**) shows the detailed analysis of the full samples. In this case, density (top) and curvature (bottom) distributions of all clusters for each cluster dimensions were compared at one time using a KS-/ WX-test (blue/red). The results indicate the dissimilarity of clusters for all cluster dimensions. As an indicator, an aggregated p-value of KS- and WX-test is determined using the weighting t-Norm-AND. The p-value (black dashed line) remains mostly below the critical p-value area (orange bar) disproving the similarity hypothesis. The corresponding values for (dis)similarity ***sim***_***M***_ = 0.01/0.68 for the density and curvature distributions were determined respectively. A closer look at the KS-/WX-Test results on curvature comparison indicates that for cluster dimensions approx. 600 nm– 800 nm the distribution is above the critical p-value area (indicating similarity). (**b**) shows the aggregated p-value of density and curvature calculated with the weighting t-Norm-AND (blue) and the confidence interval (black dashed line). A ***sim***_***M***_ = 0.36 is determined. The full-sample analysis was only, additionally performed, in order to show the full software capabilities (results are typically not used for a multilayer analysis). For such large samples, the fast bootstrap resampling is used. The graphs (**c-d**) clearly show a general dissimilarity of the samples. (**d**) depicts 100 curves–representing relative density of 100-resamples each including 2000 points (green)–used for the bootstrap based comparison of the cluster/curvature density distribution for both samples. In black, the averaged curve is represented. The detailed analysis of the bootstrapped samples (**d**) was performed as described in (**a**). (**c**) shows the statistical comparison of the cluster density (top) and curvature data (bottom), determined from the bootstrap results. For such detailed analysis (**c**), KS/WX- tests (blue/red) as well as the t-Norm-AND values (black) are plotted. Additionally, the boundaries of the confidence interval are displayed (magenta). The statistical comparison of the bootstrap derived data shows a ***sim***_***M***_ = 0.21/0.76 and ***sim***_***L***_ = 0.11/0.4 for cluster density and curvature comparison, respectively. The data on cluster density as well as curvature however, shows a general dissimilarity of the samples. A similarity for cluster curvatures for the dimension ranges of approx. 200 nm– 400 nm and approx. 600 nm– 800 nm is observed. (**e-i**) depicts the **second level analysis**. (e) shows the p-values determined by the mean-cross analysis of the average density (left)/curvature (right) values determined via bootstrapping. Herein, p-values of the mean-cross comparison (blue) and lower/upper confidential boundaries (black dashed line) are shown. The ***sim***_***M***_ = 0.01 and 0.61 and ***sim***_***L***_ = 0.005/0.1 for cluster density and curvature comparison are determined respectively. In general, the data emphasizes the dissimilarity hypothesis, however, the data for curvature comparison indicates a significant similarity for approx. 400 nm– 500 nm and >900 nm cluster density ranges. (**h**) shows the aggregated p-values for cluster curvature and density comparison (blue). The aggregation of the p-values results in ***sim***_***L***_ = 0.24, indicating a general dissimilarity. (**f**) depicts the calculated K-/H-Ripley function values for the bootstrapped data (green) for all cluster dimensions. Using H-Ripley as the crucial dimension, representative for the largest difference (clustering caused) between the sample and complete spatial randomness, can be determined (for the two images the largest difference occurs for clusters sizes of 210nm). (**g**) depicts the comparison of the K-/H-Ripley function (K-function left, H-function Right) values at the second statistical level. The p-values of the cross comparison (red), mean-cross comparison (blue) and lower/upper confidential boundaries (black dashed line) are shown. In contrast to all the data presented on single molecule clustering, the cross-comparison on the global analysis via Ripley’s function indicates sample similarity (for clustering dimensions ranging between approx. 400 nm– 1 000 nm). Similar to the individual distributions, is the aggregation of the K-/H-Ripley functions results (**i**). The respective ***sim***_***M***_ values for the Ripley’s functions comparison are 0.86 (K-/H-Ripley) and 0.55 (aggregated). The values are contrary to the previously shown results obtained on direct cluster comparison, thus a similarity is indicated. The varying result emphasizes the importance of a multilevel statistical analysis.
(TIF)

S9 Fig Detailed statistical comparison of interleukin-1β-treated and untreated platelets within a clot. Cells were labelled with Alexa647-labelled anti-CD62p-antibody. Images were taken at the edge of an artificial clot, where platelets are more sparsely distributed than within the clot. Therefore, segmentation (ROI extraction) is required. Here, we present a statistical comparative analysis of the full image, with sparsely distributed platelets and we compare the extracted regions. The full (not regionalized) sample analysis is depicted in the left column of the Fig (**a’-e’**), the ROI analysis on the right ((**a-e**); parts of the ROI analysis are included in Fig 3 in the main text). From the analysis, the advantage of segmentation (region extraction) is clearly visible. The **first level analysis** of the comparison of the KS/WX- tests presented in (**a**) (and **Fig 3E**) and (**a’**) indicates a significant similarity for full images. For ROI-based comparison, density and curvature of the clusters shows similarity solely for cluster dimensions >700 nm (***sim***_***M***_/***sim***_***L***_ = 0.47/0.31 and 0.45/0.24 for cluster density/curvature distributions, in contrast: ***sim***_***M***_/***sim***_***L***_ = 0.87/0.75 and 0.87/0.80 for a full sample). **Fig 3C** (main text) depicts two extracted regions of an untreated (upper) and IL-1β treated (lower) clot; a difference in the CD62p distribution can be observed. The divergence of the results between ROI and full image analysis is due to numerous small clusters, which are homogenously distributed in the images; in particular in the platelet-free area of the interleukin-1β treated clot. Not-cell-related signals add a significant additional cluster population, which is less significant within a ROI. For full image analysis, clusters for a specific cluster dimension originate from various regions due to hierarchical clustering, which influences the overall cluster density/curvature distribution. This is not the case for ROI-based analysis. Similar results can be observed for the **second level analysis**: the mean-cross comparison of the statistical data on cluster density and curvature show divergent results. As before, a dissimilarity for the ROI comparison is shown (***sim***_***M***_/***sim***_***L***_ = 0.07/0.07 (**Fig 3F**) and 0.91/0.50 (**b, c**) for aggregated cluster density/curvature comparison, in contrast: ***sim***_***M***_/***sim***_***L***_ = 0.54/0.2 and 0.85/0.35 for cluster density/curvature comparison on a full sample (c’)). The results are confirmed by the K-/H-Ripley’s statistics. The mean-cross analysis of all values, shown in (**d, d’**), indicates that many platelets within the extracted clot regions are sparsely distributed. Again, solely K-/H-Ripley statistics determined for the extracted ROI’s indicate a difference in CD62p protein secretion after interleukin 1β treatment (***sim***_***M***_/***sim***_***L***_ = 0.03/0.01 and 0.04/0.01 for K-/H-Ripley comparison (ROI analysis), in contrast: ***sim***_***M***_/***sim***_***L***_ = 0.94/0.78 and 0.94/0.79 for K-/H-Ripley comparison on a full sample).
(TIF)

S10 Fig Filtering of 3D dSTORM images using DBSCAN, ROI extraction, graphical representation of protein clusters. Images are reconstructed from approx. 60 000 single localisations. Images are shown before (**a**) and after (**b**) filtering using DBSCAN. Reduction from 59 393 points to 51 323 points (red: negative z values, yellow: positive z values) was achieved by using ρ = 2 and *minP* = 3. Points defined as outliers are filtered. (**c**) represents the sample image shown in (**a**) before region extraction (white box: large regions, red box: small regions). (**d**) shows the extracted region (region includes all points from original image). Cluster volume was calculated as sum of spheres (**e**), convex-hull volume (**f**) and box volume (**g**).
(TIF)

S11 Fig Comparison of different visualisation methods of clusters. Visualisation of clusters with a maximum size of 210 nm. (a) shows the 600 densest clusters of sample 1 and (b) the 600 densest clusters of sample 2. (c) depicts the triangulation-based representation of a cluster from the inset in (b). (d) shows the sphere packing-based representation of the chosen clusters. Visualisation of the primary clusters of sample 2, created with a neighbourhood radius of 250 nm. (e) depicts the largest primary clusters, (f) the triangulation-based representation of the largest cluster and (g) the sphere packing-based representation of the largest cluster. (h) shows the empirical probability density function (pdf) for density distribution of primary clusters. (i) shows the empirical pdf for curvature distribution of primary clusters. The red and blue curves in (h,i) represent the samples from (a) and (b), respectively.
(TIF)

S12 Fig Comparison of CD62p expression (mean cell intensity) of untreated and cytokine treated platelets. CD62p expression (detected as mean cell intensity (int.) of cells labelled with anti-CD62p-Alexa647) was significantly lower after 30 minutes of 10 ng/mL interleukin-1β treatment (mean: 5219 +- 1856 counts; n = 88 compared to 3565 +- 1246 counts; n = 83; power: 0.94). These data show a statistically significant lower CD62p expression for IL1-β treated cells in 2D (i.e. on single spread platelets). The boxplot depicts the median and the first and third quantile and outliers.
(TIF)

S13 Fig Comparison of randomly distributed (simulated) localisation events, platelets of different morphologies and the CD41 protein distribution in a reconstructed dSTORM image. (a) shows the distribution of CD62p labeled with Alexa647-antibody. (b) represents a randomly distributed point cloud (approx. 85 000 data points/image). (c) shows the comparison of the cluster densities distribution to Ripley’s K-function distributions for all cluster dimensions between the simulated and CD62p datasets (black/blue line for cluster density/K-function, respectively). The lines depict the aggregated p-value of KS- and WX-test results. The test based on the Ripley’s K-function classifies the samples as similar (similar for cluster sizes ranging from 5 nm to about 400 nm). In contrary, our classification estimates the samples as dissimilar, with the test based on the density distribution of clusters (similarity measure = 0). The MLP neural network classifies samples as dissimilar with a posteriori probability of 88%. (d) and (e) show a simulated polarized (randomly distributed) and unpolarised image respectively. (f) shows the comparison of clusters density/curvature distributions and Ripley’s K-functions for all cluster dimensions between the simulated polarized and simulated unpolarized datasets (black/blue line for cluster density/K-function, respectively). The lines depict the aggregated p-value of KS- and WX-test results. The test based on the Ripley’s K-function classifies the samples as dissimilar (similar for cluster sizes ranging from approx. 150 nm– 180 nm). Our classification estimates the samples as dissimilar, with the test based on the density distribution of clusters (similarity measure = 0). Three additional pairs of polarized and unpolarised samples have been compared, rendering a dissimilarity of 78.5%, 88.5% and 95.1%. (g) and (h) show reconstructed dSTORM images of Alexa647-phalloidin labelled actin skeleton of two individual platelets activated on a glass surface. The two platelets are comparable, in an early activation state. (i) depicts the comparison of the cluster densities and the Ripley’s K-function distributions for all cluster dimensions between the datasets representing platelet cytoskeleton at a similar activation stage (black/blue line for cluster density/K-function respectively). The lines depict the aggregated p-value of KS- and WX-test results. The test based on Ripley’s K-function classifies samples as strong dissimilar. Our analysis provides a correct classification of samples as similar, based on the density distribution of clusters (similarity measure = 0.86). The MLP neural network classifies samples as similar with a posteriori probability of 87%. (j) and (k) show reconstructed 3D dSTORM images of Alexa647-phalloidin labelled actin skeleton of two individual platelets activated on a glass surface. The two platelets are in two different activation states, (j) a late activation state and (k) an early activation stat. (l) shows the comparison of the cluster densities and of Ripley’s K-function distributions for all cluster dimensions between the datasets representing localisation microscopy images of platelets in early and late activation state (black/blue line for cluster density/K-function respectively). In this case both tests based on the Ripley’s K-function as well as on the cluster density distribution correctly classify the samples as dissimilar (similarity measure = 0.1). The MLP neural network classifies rather samples as dissimilar with a posteriori probability of 64%. The results clearly prove that our method is also capable to compare protein distributions that change over time induced by external factors at a single cell level. The axial range is represented by two colors: blue is below the focal plane and yellow above. The axial range is ± 500 nm.
(TIF)

S14 Fig **Comparison between the CD62p distribution in interleukin treated clots** (large clot on the left side and a chosen region of interest on the right). First level statistics yields for the two samples similar cluster densities for all cluster dimensions (***sim***_***M***_ = 0.85) and for cluster curvatures (***sim***_***M***_ = 0.32). Similar values are obtained for a comparison of aggregated density and curvature values (***sim***_***M***_ = 0.76 and ***sim***_***M***_ = 0.85). Dissimilarity for the H-/K-Ripley analysis can be observed (***sim***_***M***_ < 0.2). An MLP value of 0.66 proves the similarity of the samples. The detailed analysis directly shows the crucial dimensions and parameters, which best describe the differences between both samples. The results prove that the differences between large clusters as well as small ROIs (size ranges of individual cells) can be very precisely determined.
(TIF)

S1 Table Statistical comparison (p-values) of CD41/CD62p pairwise comparisons. The table displays all statistical p-values determined from several chosen pairwise compared distributions of CD41/CD62p proteins in clots. Samples 1–4 are samples where CD41 has been stained with Alexa488-labelled anti-CD41-antibody and samples 5–6 are samples where CD62p was stained with Alexa647-labelled anti-CD62p-antibody.
(XLSX)

S2 Table Statistical comparison (p-values) of CD62p upon IL-1β treatment. The table displays all statistical p-values determined from several chosen pairwise compared distributions of CD62p proteins in IL-1β treated/untreated clots. Samples 1–3 display the comparison of CD62p from IL-1β untreated samples (stained with Alexa488-labelled anti-CD41-antibody) and samples 4–5 show the comparison of CD62p from IL-1β treated samples.
(XLSX)
