# Supplementary material for: Statistical analysis of 3D localisation microscopy images for quantification of membrane protein distributions in a platelet clot model
Source: PLoS Comput Biol. 2020 Jun 30;16(6):e1007902. doi: 10.1371/journal.pcbi.1007902 (PMC7384682; doi:10.1371/journal.pcbi.1007902)
Supplement: S1 Methods — (PDF) [file pcbi.1007902.s001.pdf]

Statistical analysis of 3D localisation microscopy images for quantification of membrane protein distributions in a platelet clot model

Sandra Mayr, Fabian Hauser, Sujitha Puthukodan, Markus Axmann, Janett Göhring, Jaroslaw Jacak

Glass coating

Glass slides were washed with distilled water, 70% iso-propanol and again with distilled water before being dried with a stream of nitrogen-gas. In order to increase protein adhesion, glass substrates were plasma-cleaned (30 minutes with oxygen plasma and 45 minutes with argon plasma, 100 W (Diener electronic, Ebhausen, Germany)). Subsequently, slides were coated with 50 µg/mL rat collagen (Sigma Aldrich, Vienna, Austria) to mimic the extracellular matrix. Prior to clot preparation, the protein-coated surface was washed with Phosphate Buffered Saline (PBS) and dried with nitrogen gas.

Human platelet concentrates

All human blood samples were collected during routine thrombophoresy in accordance with the strict policies of the Red Cross Transfusion Service Linz (Austria). All blood donors signed their informed consents that residual blood material could be used for research and development purposes. All experimental protocols were approved by and carried out in collaboration with the Red Cross Blood Transfusion Service Linz. Single donor platelet concentrates were provided by the Red Cross Blood Transfusion Service Linz. Platelet concentrates were prepared with an automated cell separator (Trima Accel Automated Blood Collection System, TerumoBCT, Lakewood, CA, USA) by apheresis during routine thrombophoresy. Platelets were separated from whole blood by centrifugation and diluted in 35% plasma, 65% platelet additive solution SSP+ (Macopharma, Mouvaux, France), and ACD-Anticoagulant (Haemonetics anticoagulant citrate dextrose solution, Haemonetics, Braintree, MA,USA) during the transfer into Trima Accel storage bags. 2 mL of the platelet concentrate (approx. 1×10^6 platelets/mL) were transferred into a storage bag and immediately transported to the laboratory in a polystyrene box (in order to minimize temperature variations). All experiments were performed within 24 h after platelet isolation – cells were stored under constant agitation in a climatic chamber that was tempered to 22 °C.

IL-1ß treatment

Platelets were incubated with 10 ng/mL of recombinant interleukin 1 beta (IL-1β, Sigma-Aldrich, Vienna, Austria) for 30 minutes at 22 °C. Subsequently, the platelets were centrifuged and resuspended in PBS.

Platelet clot formation

$1x10^8$ platelets were centrifuged at 500xg for 10 minutes to remove the anticoagulant storage solution from the concentrate. After resuspension in PBS, cells were gently mixed with 2.5 mg/mL fibrinogen (from bovine plasma, Sigma, Saint Louis, USA) and 0.2 U/mL thrombin (kind gift from LBI Trauma Care Vienna, Austria) in 40 mM $CaCl_2$ and clots (20 µL contain $1x10^7$ platelets) were pipetted onto either plain or collagen-coated glass. Clots were air-dried for 30 minutes at room temperature prior to fluorescence labelling.

Fluorescence labelling of platelet clots

Clots were fixed with 4% paraformaldehyde in PBS for 20 minutes at room temperature. After washing, blocking was performed in 10% albumin from chicken egg white (Sigma-Aldrich, Vienna, Austria) and cells were stained overnight using the following antibodies (experiment-dependent): mouse monoclonal anti-CD62p antibody conjugated to Alexa647 (1 µg/mL, BioLegend, San Diego, USA) and mouse monoclonal anti-CD41 antibody conjugated to Alexa488 (1:100 dilution, EXBIO Praha, Vestec, Czech Republic).

Fluorescence microscopy and 3D dSTORM image acquisition

Images were acquired using a modified Olympus IX81 inverted epi-fluorescence microscope with an oil-immersion objective (PlanApo N 60x 1.42 NA, Olympus, Vienna, Austria). The sample was fixed on a XYZ piezo stage (PI Mars; P-562, Physical Instruments) with nanometer precision combined with a coarse mechanical stage with a travel range of 1 cm x 1 cm (Hybrid, JPK Instruments, Berlin,

Germany). A tube lens with an additional magnification of 1.6 was used to achieve a final imaging magnification of 96 (corresponding to a pixel size in the image plane of 167 nm). Platelets were illuminated with a 642 nm laser light from a diode laser (Omicron-laserage Laserprodukte GmbH, Phoxx 642, Rodgau-Dudenhofen, Germany), a 532 nm laser light from a solid-state laser (diode-pumped, Cobolt AB, Solna, Sweden), a 488 nm laser light from a solid-state laser (diode-pumped, Toptica Photonics, Graefelfing, Germany), and a 405 nm laser light from a diode laser (Insaneware, Gladbeck, Germany). The fluorescence signal was detected using an Andor iXonEM+ 897 (back-illuminated) EMCCD camera (16 µm pixel size). The following filter sets were used: dichroic filter (ZT405/488/561/640rpc, Chroma, Olching, Germany), emission filter (446/523/600/677 nm BrightLine quad-band band-pass filter, Semrock, Rochester, NY, USA), and additional emission filters: ET 700/75 M, Chroma Technology GmbH, Olching, Germany; ET 525/50 M, Chroma Technology GmbH, Olching, Germany; ET 595/50 M, Chroma Technology GmbH, Olching, Germany. For 3D measurements, a cylindrical lens (f=500 mm; Thorlabs, Newton, USA) was placed into the emission path of the microscope.

3D dSTORM Imaging Protocol

Single-molecule photo-switching of the rhodamine dye Alexa Fluor 488 and the cyanine dye Alexa Fluor 647 was performed in a buffer optimized for both fluorophores in order to image both channels without the need for buffer exchange. The buffer containing OxEA, as described by Nahidiazar et al. [1], was applied to the cells immediately prior to the fluorescence microscopy measurements. For imaging, the sample was illuminated for 20 ms with 1.2 kW/cm^2 excitation intensity (647 nm) and 3.3 kW/cm^2 excitation intensity (488 nm) (both frame rates: 25 images/s), respectively. During camera readout, the sample was illuminated with a 405 nm laser light (10 ms at 100 W/cm^2) to recover fluorophores from the singlet ground state. The deformation characteristics introduced by the cylindrical lens are experimentally determined via a calibration step before each experiment using immobilized fluorescent diffraction-limited beads; this allows for determination of the axial position of a single-molecule from the deformation of its signal (3D dSTORM). Typically, we acquired

10 000 frames including approx. 75 000 – 500 000 single-molecule signal events. Typically, single-molecule signal levels of 1676 ± 1245 and 1263 ± 637 photons were derived for Alexa 647 and Alexa 488, respectively (illumination time = 20 ms). All illumination protocols were performed with custom-written acquisition software.

3D dSTORM fitting of single-molecules and visualization

The previously presented dSTORM analysis workflow [2] was adapted to analyse the axial position of single molecules depending on the point spread function (PSF) deformation. We used a two-dimensional Gaussian function as model to reduce the calculation time (instead of an integrated Gaussian function):

$$f(x,y,\vec{\theta}) = \theta_b + \theta_p \, e^{-0.5\left[\frac{(x-\theta_x)\cos\theta_\varphi + (y-\theta_y)\sin\theta_\varphi}{\sigma_x(\theta_z)}\right]^2 - 0.5\left[\frac{-(x-\theta_x)\sin\theta_\varphi + (y-\theta_y)\cos\theta_\varphi}{\sigma_y(\theta_z)}\right]^2}$$

A calibration was performed to assign the axial position of single molecules to a specific elliptical PSF aberration. Calibration was acquired by moving the sample (TetraSpeck beads, ThermoFisher, Vienna, Austria) in the axial direction with a step size of 10 nm over 2 µm. Each frame is analysed and multiple PSFs are fitted with a two-dimensional Gaussian function. The widths $\sigma_{x,y}$ of the calculated PSFs are smoothed using a cubic-B-spline[3] and assigned to the defined axial position. The fitting model for the z-dependant size change of $\sigma_{x,y}$:

$$\sigma_{x,y}(z) = \sigma_0 \sqrt{1 + \left(\frac{z-c}{d}\right)^2 + A\left(\frac{z-c}{d}\right)^3 + B\left(\frac{z-c}{d}\right)^4}$$

From this model, we calculate the depth of focus d as well as the axial distance Δl, which is the mean distance from the minima to the intersection (focus) of the curves. These parameters are needed to calculate positional accuracy. The direct fitting of the axial position of the molecules strictly depends on the set of initial parameters. Hence, an appropriate guess of the starting parameters is required. For estimation of the initial parameters, we fit two simple one-dimensional Gaussian functions in the horizontal and vertical profile of the PSF. The determined widths of the Gaussian functions are used

to calculate the z-position by using a linear regression model ($\varepsilon(z) = \sigma_x(z) - \sigma_y(z)$) of the calibration curves. Additionally, parameters such as position, peak- and background-intensity are determined. The model is minimized using the Double Dogleg [4] algorithm. In the next step, the positional accuracy is calculated. The equations are based on the Thompson & Mortensen formulas [5], but were expanded to handle astigmatism as well as the axial accuracy [6]. In order to speed up the visualisation process, single molecules are sorted axially and drawn using false colours with a circle of pseudo-Gaussian decrease of the alpha colour channel.

Drift correction

The core of this algorithm is to determine a displacement vector $\vec{d}$ that minimizes the pairwise distance between two position sets S_A and S_B, where S_A is fixed and S_B is mobile (positions plus displacement vector). The similarity of two positions of individual points is given by a Gaussian distribution with a mean value of the Euler distance between these positions and a variance value of the average positional accuracy over all positions of points. The cost function is then the summation of all similarity values. Finally, the goal is to find a displacement vector $\vec{d}$ (drift vector) that maximizes the cost function $CF(\vec{d})$.

$$CF(\vec{d}) = \frac{1}{2\pi\sigma^2}\sum_{j}^{M}\sum_{i}^{N}\exp\left(-\frac{\left\|S_{A,i} - (S_{B,j} + \vec{d})\right\|^2}{2\sigma^2}\right)$$

At the beginning, the algorithm needs to build up a basis. Within just a few frames and at a high temporal resolution, there should be almost no drift. The basis is filled with positions from initial frames until a defined limit of points is reached. Afterwards, the remaining frames are grouped into n-sets, which have at least a defined number of points. Typically, 150 positions of points are more than enough for the basis (fixed set) and 50 positions of points for each of the mobile sets. Beginning from the first mobile set and compared to the basis set, the displacement (drift) vector is calculated. This vector is then applied to each position of the current mobile set and the containing points are added to the basis set. Therefore, for each calculated displacement vector, the basis will be

expanded. The drift compensation process is repeated until all mobile sets are corrected. In case of a large displacement, a new drift vector is calculated. Herein the present corrected positions are subtracted from the original positions of the processed points and an average distance vector is calculated. The time-dependent drift vectors are then smoothed using cubic-B-splines. Finally, the smoothed and interpolated drift curves can be applied to the original molecules.

Outlier filtering

Points are classified as core points or outlier points according to the following algorithm: Point p is called a core point in cases when at least a number of $minP$ points are located within the distance ρ. Distance ρ is the given maximum radius of the p neighbourhood. Thus, any point q is directly accessible from p, if point q is within the distance ρ to the point p. In general, a point q is called reachable from the point p, in case there is a path $p_1, \dots, p_n$ with $p_1 = p$ and $p_n = q$; where each p_{i+1} is directly reachable from p_i. All points that are not reachable from any other point are classified as outliers. Hence, all points in a cluster (called primary clusters) are mutually 'density-connected' to each other; in case a point is reachable from any cluster point, it is also a part of the cluster [7-11]. For clustering, DBSCAN requires two parameters:

- ρ - The neighbourhood radius
- $minP$ - The minimum number of points (neighbours) required to form a dense region

First, the algorithm starts at an arbitrary point and calculates this point's ρ–neighbourhood. If the point has sufficient neighbouring points ($\geq minP$), a cluster is started. Otherwise, the point is set as an outlier. A value of $minP$ that is too large eliminates points even inside a dense cloud. The minimum number of neighbours required to form a cluster depends on the specified filtering accuracy (typically *minP* = 4 or 5, including *p*). The quality of the filtering depends strictly on the size of the chosen neighbourhood radius ρ. DBSCAN in our platform uses the Euclidean 3D-distance function for calculation of radius ρ [8-10, 12]. The appropriate radius for the sample is estimated as follows: Let us assume that nn (nearest neighbours vector) is a vector of minimal distances to the

nearest neighbours for each point $(p_1, \dots, p_n)$ in the point cloud. ρ is calculated as the average value of the vector nn multiplied by the α-filtering accuracy factor: $\rho = \alpha \cdot mean(nn)$. A higher coefficient α yields bigger primary clusters (Note: Secondary clusters are all points that are not within the defined parameters of the DBSCAN and are considered as outliers). The coefficient α is chosen experimentally and has values between 1 and 3 (typically: $\alpha = 1.5$). The calculation of the vector nn can be very time-consuming (especially for $> 200\,000$ points). Therefore, for large samples, it is recommended to estimate average values of minimum distances between neighbours. The estimations of the average value of the vector nn are carried out using K-data-resampling with randomly selected M points (M ~ 30 000 or 40 000). The radius *mean(nn)* can be estimated as the average of the average minimum distances to the nearest neighbours for each resampled data, i.e. $mean(nn) \approx mean(mean(nn_i | i = 1, \dots, K))$. The points in low-density regions (points with far away neighbours) are marked as outliers. As a result, the points with a small number of neighbours are discarded (~~S7~~S10-**a,b Fig**).

Extraction of regions-of-interests in samples

To analyse isolated 3D clouds of points, regions-of-interests (ROIs) can be automatically extracted. When the clouds of points are cumulated in regions within a single image, these regions can be combined to ROIs (accordingly cut out of the image). For ROI extraction from the samples, a DBSCAN algorithm is also used. In order to extract plausible regions, the factor α should be increased to ~ 2.5 and the number of nearest neighbours ($minP$) should be minimal, i.e. $minP = 3$. Each region is saved as a separate subsample (~~S7~~S10-**c,d Fig**).

Simulation of random samples

Simulated samples with a random distribution have to reflect the statistical structure of the real sample. For this purpose, the simulated data set is generated in two stages:

In the first stage, we split the real data sample into clusters with a diameter limited by a multiple (factor 2) of the cluster dimension and obtained via the maximum Ripley H function. Thus, the

diameter is equal to twice the value of the cluster dimension at maximum Ripley H function value. For these centroid sets, empirical probability distribution function (ECDF) of their locations in 3D space is determined. Further, for each cluster with a number of points greater than 5 the parameters for the Poisson distribution are estimated.

In the second stage, random 3D-positions of cluster-centroids using the empirical distribution of ECDF obtained in step 1 are generated. Hence, for each random centroid 3D-position, we assign a randomly selected (without repetition) index of the original cluster. Next, we generate new cluster points based on the estimated Poisson distribution for the assigned cluster index. The number of generated points is equalized to the number of points in the allocated original cluster by addition of noise points from a uniform distribution.

References

1. Nahidiazar L, Agronskaia AV, Broertjes J, van den Broek B, Jalink K. Optimizing Imaging Conditions for Demanding Multi-Color Super Resolution Localization Microscopy. PLOS ONE. 2016;11(7):e0158884. doi: 10.1371/journal.pone.0158884.
2. Mayr S, Hauser F, Peterbauer A, Tauscher A, Naderer C, Axmann M, et al. Localization Microscopy of Actin Cytoskeleton in Human Platelets. International Journal of Molecular Sciences. 2018;19(4):1150. PubMed PMID: doi:10.3390/ijms19041150.
3. Proppert S, Wolter S, Holm T, Klein T, van de Linde S, Sauer M. Cubic B-spline calibration for 3D super-resolution measurements using astigmatic imaging. Optics express. 2014;22(9):10304-16. doi: 10.1364/OE.22.010304.
4. Dennis JE, Mei HHW. Two new unconstrained optimization algorithms which use function and gradient values. Journal of Optimization Theory and Applications. 1979;28(4):453-82. doi: 10.1007/BF00932218.
5. Thompson RE, Larson DR, Webb WW. Precise nanometer localization analysis for individual fluorescent probes. Biophysical journal. 2002;82(5):2775-83. doi: 10.1016/S0006-3495(02)75618-X. PubMed PMID: 11964263.
6. Rieger B, Stallinga S. The Lateral and Axial Localization Uncertainty in Super-Resolution Light Microscopy. Chemphyschem : a European journal of chemical physics and physical chemistry. 2014;15(4):664-70. doi: doi:10.1002/cphc.201300711.
7. Ester M, Kriegel H-P, Sander J, Xu X. A density-based algorithm for discovering clusters a density-based algorithm for discovering clusters in large spatial databases with noise. Proceedings of the Second International Conference on Knowledge Discovery and Data Mining; Portland, Oregon. 3001507: AAAI Press; 1996. p. 226-31.
8. Sander J. Generalized Density-Based Clustering for Spatial Data Mining: Herbert Utz Verlag, München; 1998.
9. Sander J, Ester M, Kriegel H-P, Xu X. Density-Based Clustering in Spatial Databases: The Algorithm GDBSCAN and Its Applications. Data Mining and Knowledge Discovery. 1998;2(2):169-94. doi: 10.1023/a:1009745219419.

10. Deschout H, Shivanandan A, Annibale P, Scarselli M, Radenovic A. Progress in quantitative single-molecule localization microscopy. Histochemistry and cell biology. 2014;142(1):5-17. Epub 04/20. doi: 10.1007/s00418-014-1217-y. PubMed PMID: 24748502.
11. Veeraraghavan R, Gourdie RG. Stochastic optical reconstruction microscopy-based relative localization analysis (STORM-RLA) for quantitative nanoscale assessment of spatial protein organization. Molecular biology of the cell. 2016;27(22):3583-90. doi: 10.1091/mbc.E16-02-0125. PubMed PMID: 27307586.
12. Veeraraghavan R, Gourdie RG. Stochastic optical reconstruction microscopy–based relative localization analysis (STORM-RLA) for quantitative nanoscale assessment of spatial protein organization. Molecular Biology of the Cell. 2016;27(22):3583-90. doi: 10.1091/mbc.E16-02-0125. PubMed PMID: PMC5221590.
