# Supplementary figures and images for: Statistical analysis of 3D localisation microscopy images for quantification of membrane protein distributions in a platelet clot model
Source: PLoS Comput Biol. 2020 Jun 30;16(6):e1007902. doi: 10.1371/journal.pcbi.1007902 (PMC7384682; doi:10.1371/journal.pcbi.1007902)

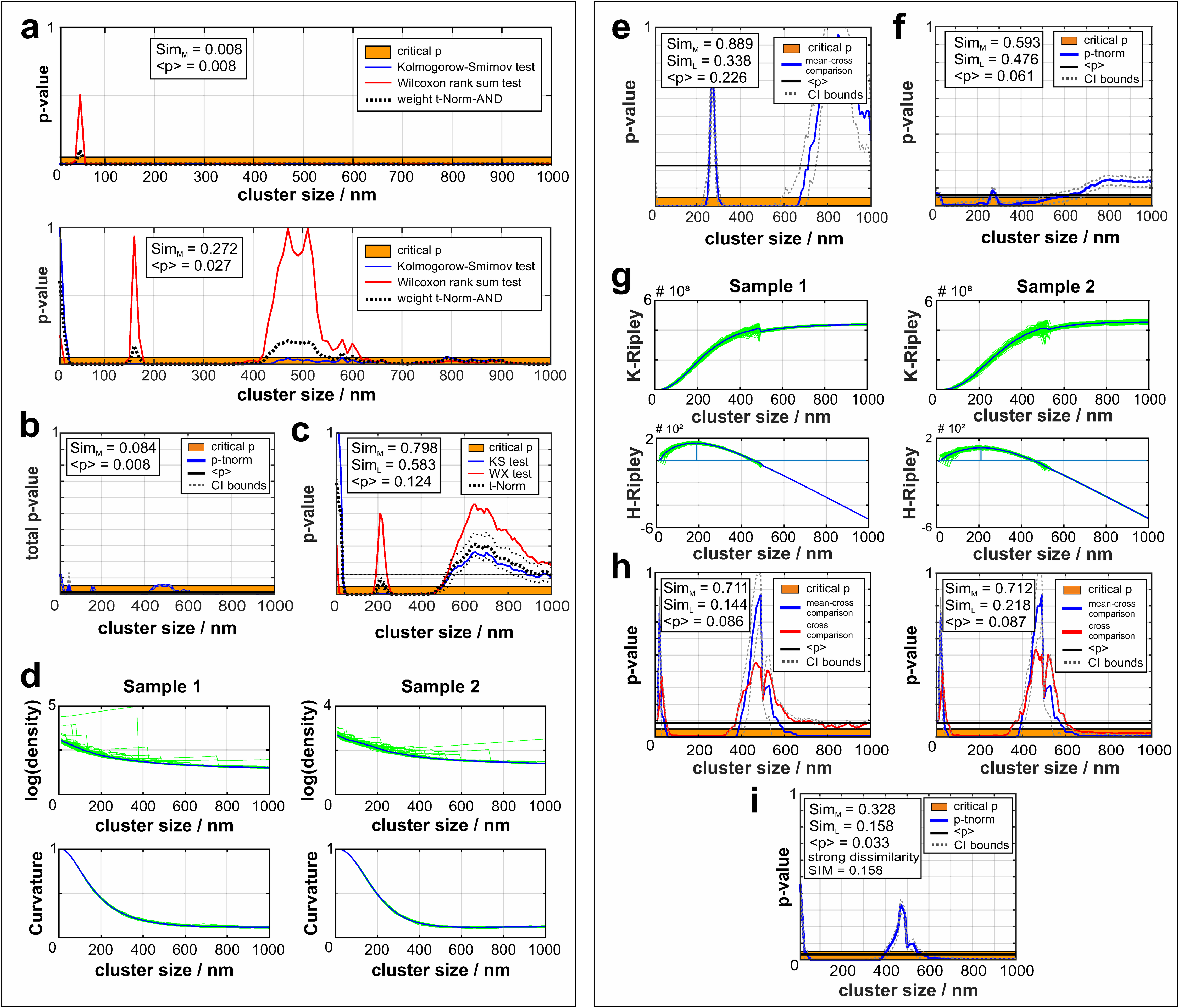

Supplement: S1 Fig — This figure shows the additional results regarding the statistical comparison for the two samples presented in Fig 2 (sample 1 has > 85 000 points and sample 2 has > 93 000 points). (a) shows the direct comparison of the full samples (all localisations) via a KS-/WX-test of the cluster densities (top) and curvatures (bottom). (b) shows the aggregated KS-/WX-test p-values of curvature calculated with t-norm-AND operator of the results represented in (a). (c) displays the values of the KS-/WX-test and their aggregation via t-norm-AND operator determined from the cluster curvature distributions received from the bootstrap resampling (comparable to Fig 2C). (d) shows the bootstrapping results for cluster density and curvature (log, and normal scale). Herein, 100 resamples (all including 8 000 points) were plotted. Using the bootstrap method allows us to gain multiple testings required for the second level analysis. (e) presents a part of the 2nd-level analysis and shows results on mean–cross comparison of the density and curvature distributions of resampled image data, respectively. (f) presents the aggregated p-value for curvature and density comparison data at both analysis levels. (g) displays the results of the K-/H-Ripley functions for all bootstrap samples. (h) shows the KS-/WX-test results of the K-/H-Ripley data derived from both samples. On the left, the comparison of the K-functions values and on the right the comparison of the H-functions are presented. (i) shows the aggregated p-value for comparisons of K-/H-Ripley data. (TIF) [file pcbi.1007902.s002.tif]

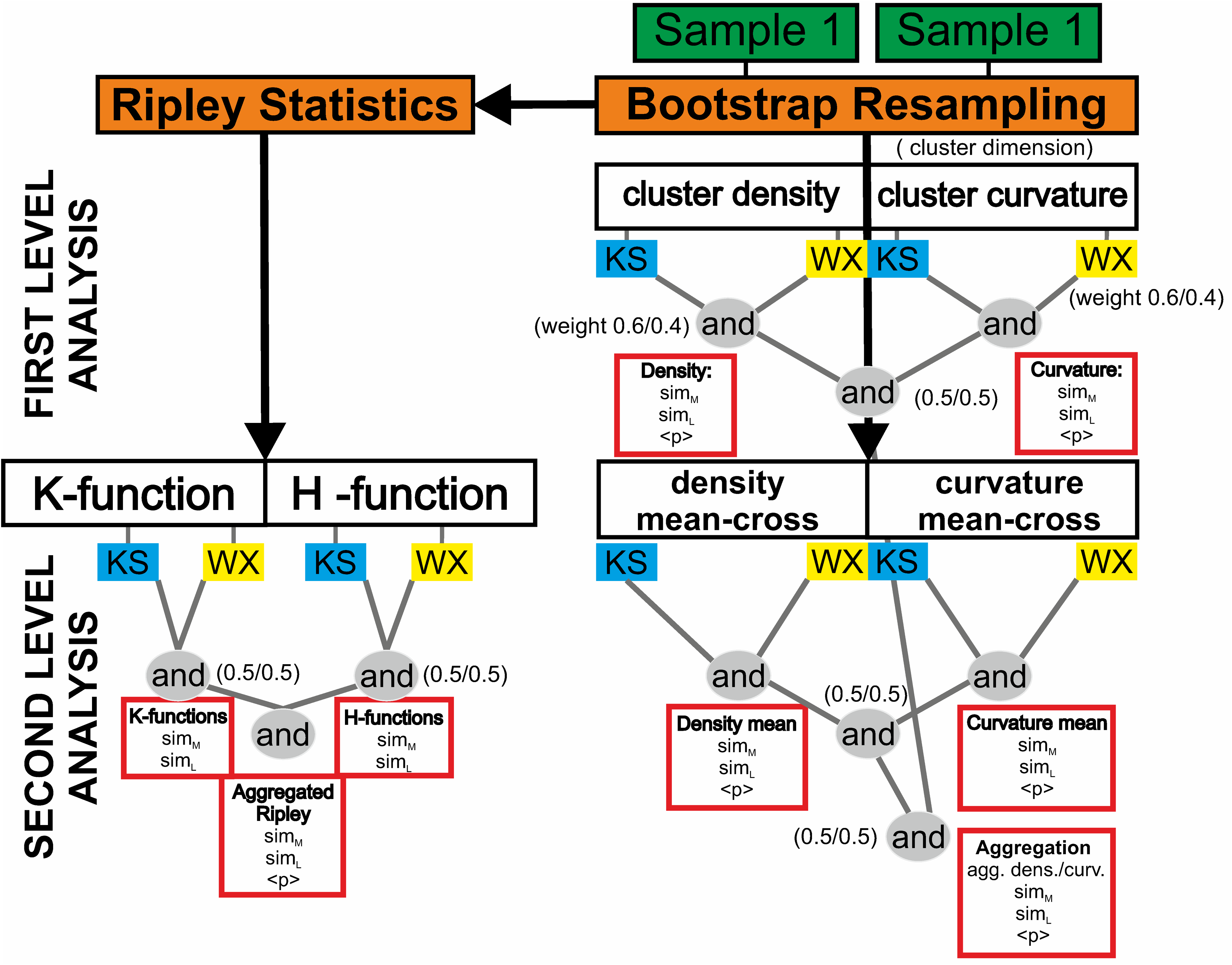

Supplement: S2 Fig — The figure depicts the flow diagram of the comparative analysis, including aggregation of parameters. In the first step, the sample data (full or bootstrap-resampled data of both samples) are clustered hierarchically for cluster dimensions (size) ranging from 10 nm– 1 000 nm. In the first level analysis of the pairwise resampled data, the Kolmogorov-Smirnov (KS)-/Wilcoxon (WX)-tests (relative density distribution/ curvature distribution) for each cluster dimension are performed. The KS- and WX-test results for comparison of cluster densities and curvatures of both (pairwise) samples are further aggregated using the t-norm-AND operator for all cluster dimensions. The results of p-value aggregation for density and curvature are once more aggregated, giving the average result of the comparison on the first statistical level. Next, the second statistical level analysis is performed. Here, the averaged density and curvature for each cluster dimension and resample are used to perform the mean-cross analysis between them. The mean-cross comparison uses KS-/WX-tests for a comparison between distributions of mean clusters density of all resamples from sample 1 and all individual density distributions of resamples from sample 2. Hence, an aggregation (t-norm AND operator) of the mean-cross KS/Wilcoxon-test results (p-values) for density and curvature for all cluster dimension gives a global p-value of similarity hypothesis. In parallel, in the second level analysis the K-/H-Ripley functions for all cluster dimensions are calculated. Hence, the mean-cross comparison on the K-/H-Ripley functions values, (same as for mean density/curvature distributions) is performed. The KS-/WX-Test results on comparison of the K-/H-Ripley function values are combined with t-norm AND, forming an additional set of parameters. (TIF) [file pcbi.1007902.s003.tif]

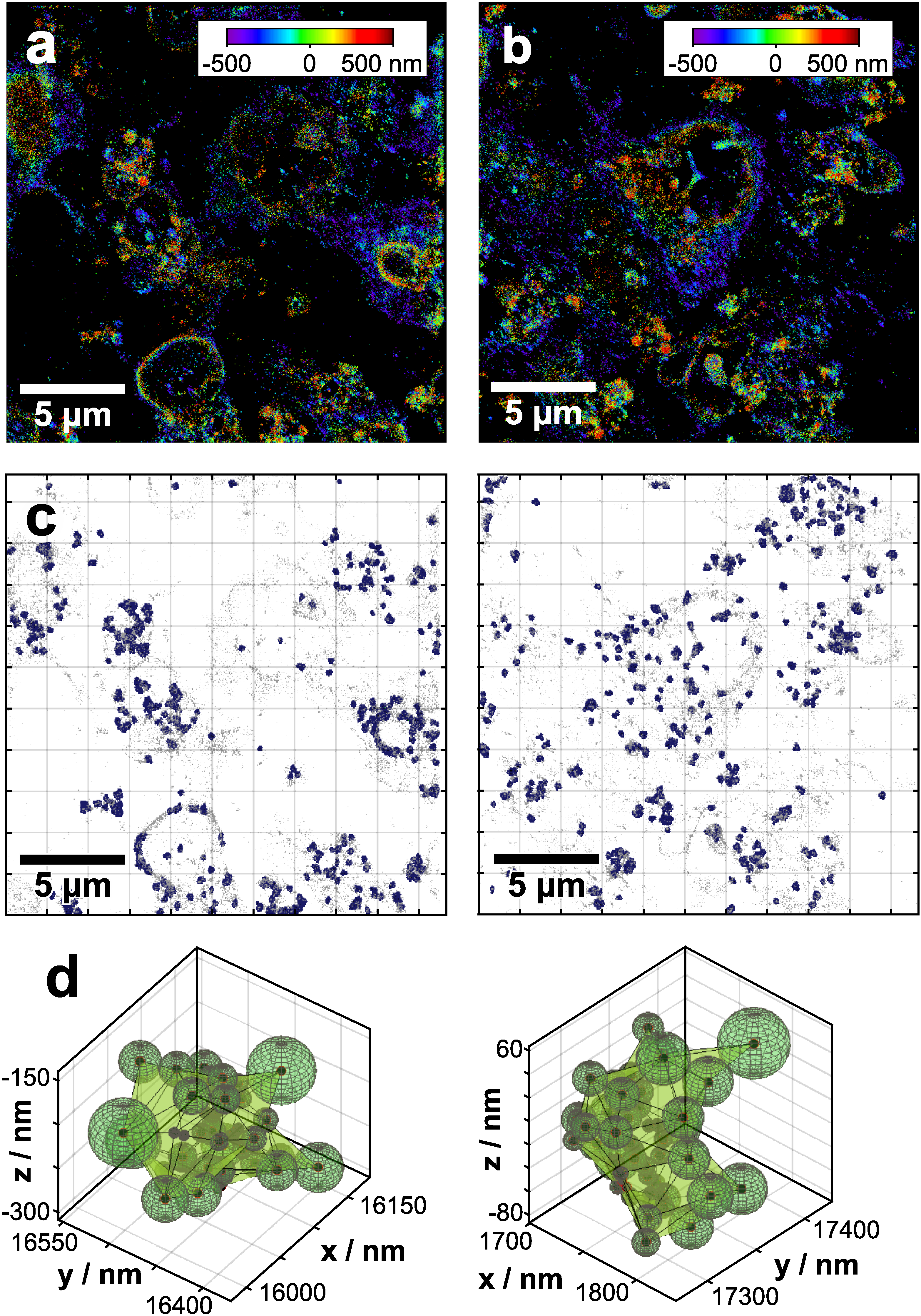

Supplement: S3 Fig — CD62p was stained with Alexa647-labelled antibody. Both reconstructed images are depicted in (a) and (b) (samples include > 93 000 and >120 000 points, respectively). (c) shows an image of selected clusters (600 clusters per image) with a cluster dimension of 180 nm. For cluster representation, we used the Delaunay-triangulation method. (d) depicts two randomly chosen clusters. (TIF) [file pcbi.1007902.s004.tif]

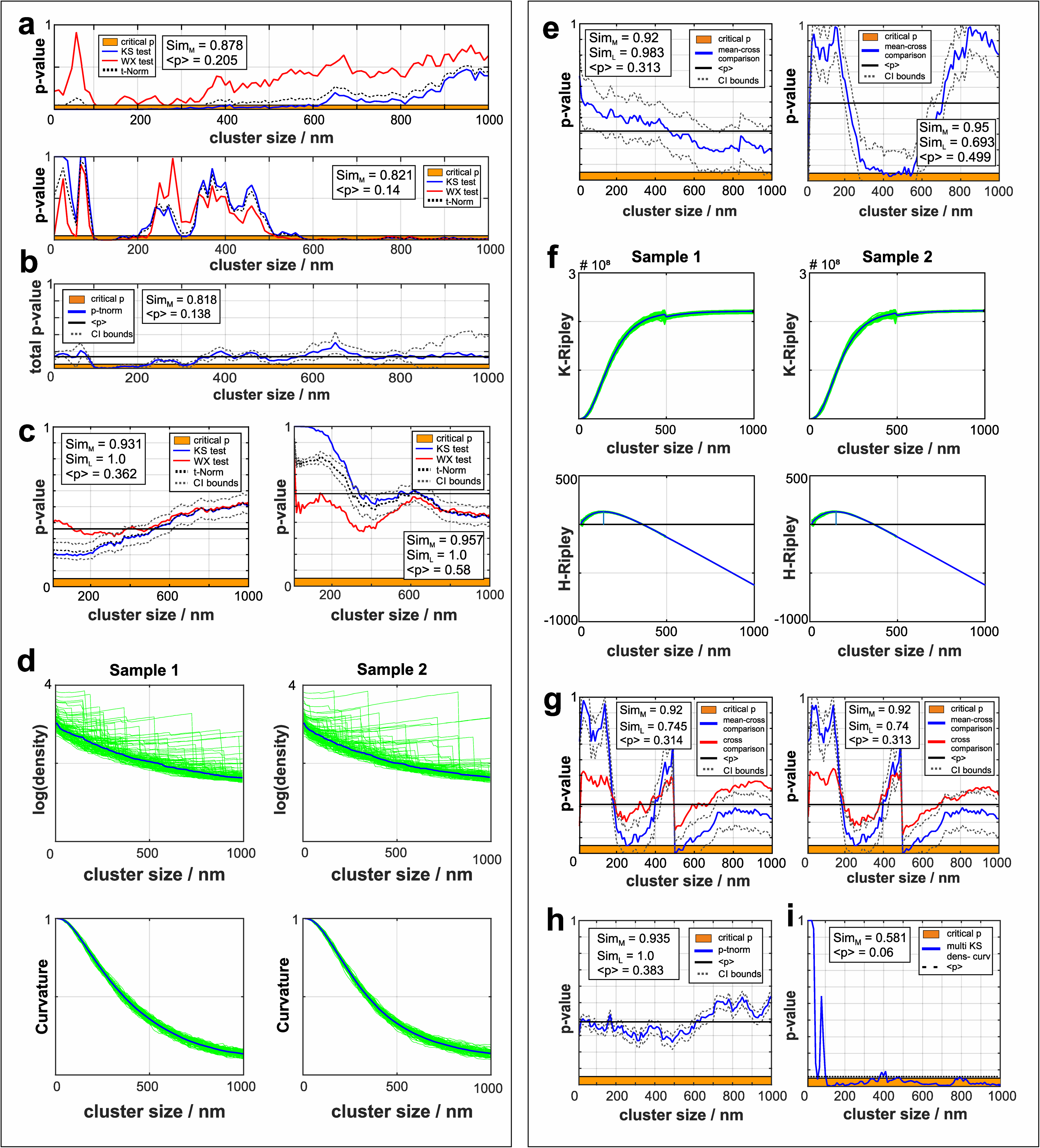

Supplement: S4 Fig — First level analysis is depicted in (a-c). (a) shows the detailed analysis of the full samples. Density and curvature distributions of all clusters for each cluster dimension were compared at one time using a KS-/WX-Test. The results indicate a similarity of clusters for all cluster dimensions. As an indicator, an aggregated p-value is determined using the weighting t-Norm-AND. The p-value (black dashed line) remains above the critical p-value area (orange bar) proving the similarity hypothesis for all cluster dimensions. The corresponding similarities are simM = 0.87/0.82 for density and curvature, respectively. (b) depicts the aggregated p-value calculated with the weighting t-Norm-AND (blue) and confidence interval (black dashed line). A value of simM = 0.82 is determined. The full-sample analysis does not give the full parameter range of the analysis, just a brief overview on cluster density and curvature similarity. (d) depicts 100 curves–representing relative density of 100-resamples each including 2000 points (green)–used for the bootstrap based comparison of the cluster/curvature density distribution for both samples. In black, the averaged values for all dimensions are represented. The detailed analysis for the bootstrap data shown in (d) was performed similarly to the analysis in (a). (c) shows the KS-/WX-test compared data regarding cluster density and curvature for all given cluster dimensions. As for the detailed analysis (d), the KS/WX-tests (blue/red) as well as the t-Norm-AND aggregated p-values are plotted (black). Additionally, the boundaries of the confidence interval are displayed (magenta). The comparative analysis of the bootstrap derived data shows a strong similarity between the samples (simM = 0.93 for cluster density, simM = 0.95 for curvature). (e-i) depict the second level analysis. (e) shows the generalized density/curvature p-values determined via a mean-cross comparison. Herein, p-values of the mean-cross comparison (blue) and lower/up [file pcbi.1007902.s005.tif]

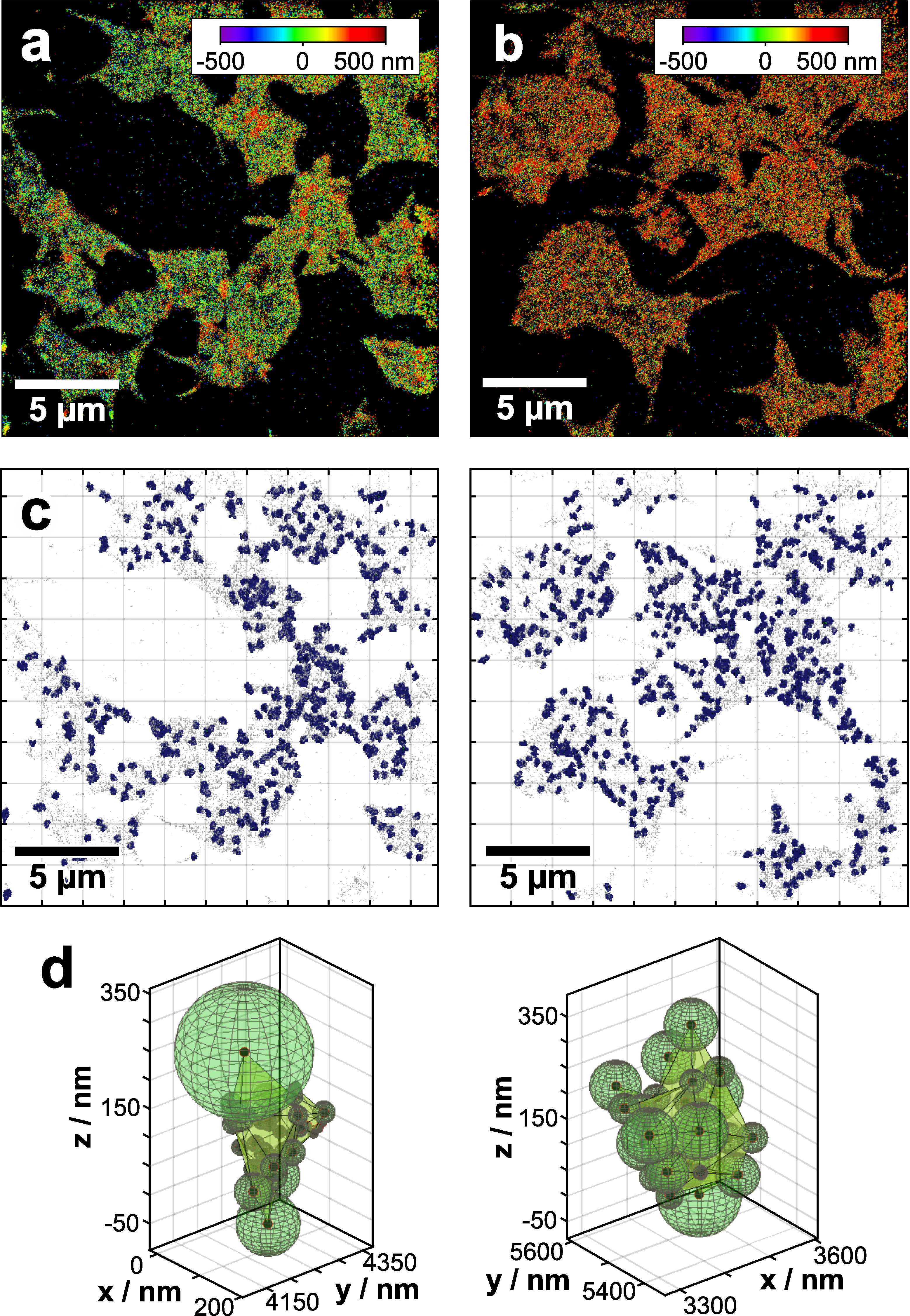

Supplement: S5 Fig — Detailed statistical comparison of CD41 cluster distributions within two clots. In both samples, CD41 was stained with an Alexa488-labelled antibody. Both reconstructed images (include >158 000 and >142 000 points, respectively) are depicted in (a) and (b). (c) shows images of the clusters with the cluster dimension of 245 nm (600 clusters in each image). For cluster representation, we used the Delaunay-triangulation method. (d) depicts two randomly chosen clusters. (TIF) [file pcbi.1007902.s006.tif]

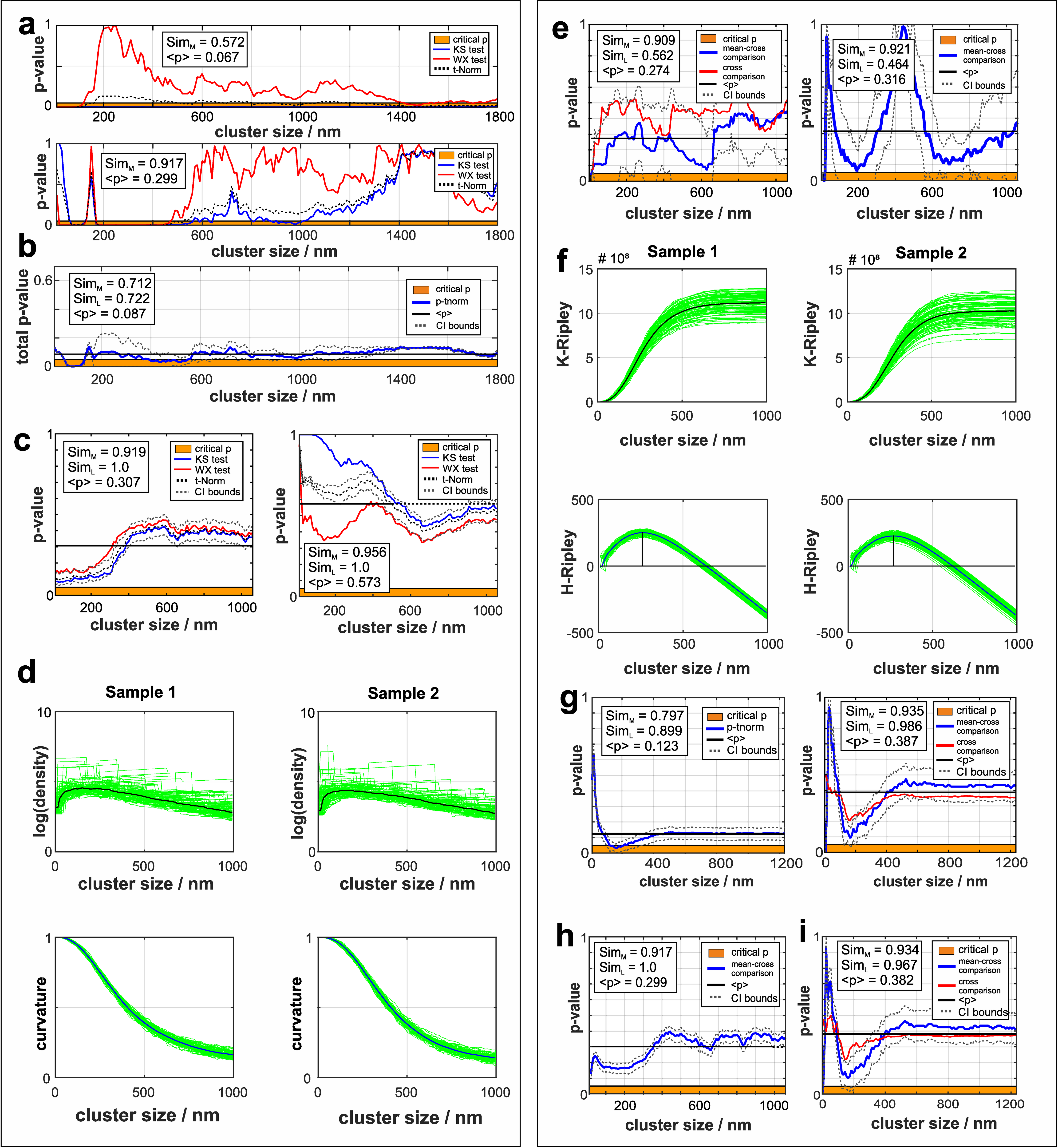

Supplement: S6 Fig — (a) shows the detailed analysis of the full samples (both samples reduced to 60 000 points) density (top) and curvature (bottom). In this case, density and curvature distributions of clusters for each cluster dimensions were compared at one time using the KS-/WX-test (blue/red). The results indicate a similarity of samples for all cluster dimensions. As an indicator, an aggregated p-value is determined using the weighting t-Norm-AND. The p-value (black dashed line) remains mostly above the critical p-value area (orange bar) proving the similarity hypothesis. The corresponding similarities are simM = 0.57/0.91 for the density and curvature distributions respectively. Remarkably, the density distribution shows a lower similarity compared to the curvature. A closer look at the comparison of the density distributions shows, that for small cluster dimensions between 10 nm and 150 nm as well as for large cluster dimensions ranging from approx. 1 400 nm– 1 800 nm, the distributions are within the critical p-value area. (b) depicts the aggregated p-value (density and curvature) calculated with the weighting t-Norm-AND (blue) and the confidence interval (black dashed line). A simM = 0.71 is determined. For such large samples, a faster bootstrap-resampling is used. (d) depicts 100 curves–representing relative density of 100-resamples each including 2 000 points (green)–used for the bootstrap based comparison of the cluster/curvature density distribution for both samples. In black, the averaged curve is represented. The detailed analysis (first level statistics) of the bootstrapped samples (d) was performed using similar statistics as used in (e). (c) shows the detailed (first level statistic) comparison of the distributions of cluster density (top) and curvature (bottom) for all given cluster dimensions (sizes). For this analysis, the KS/WX- tests (blue/red) as well as the t-Norm-AND values are plotted (black). Additionally, the boundaries of the confidence interval are displ [file pcbi.1007902.s007.tif]

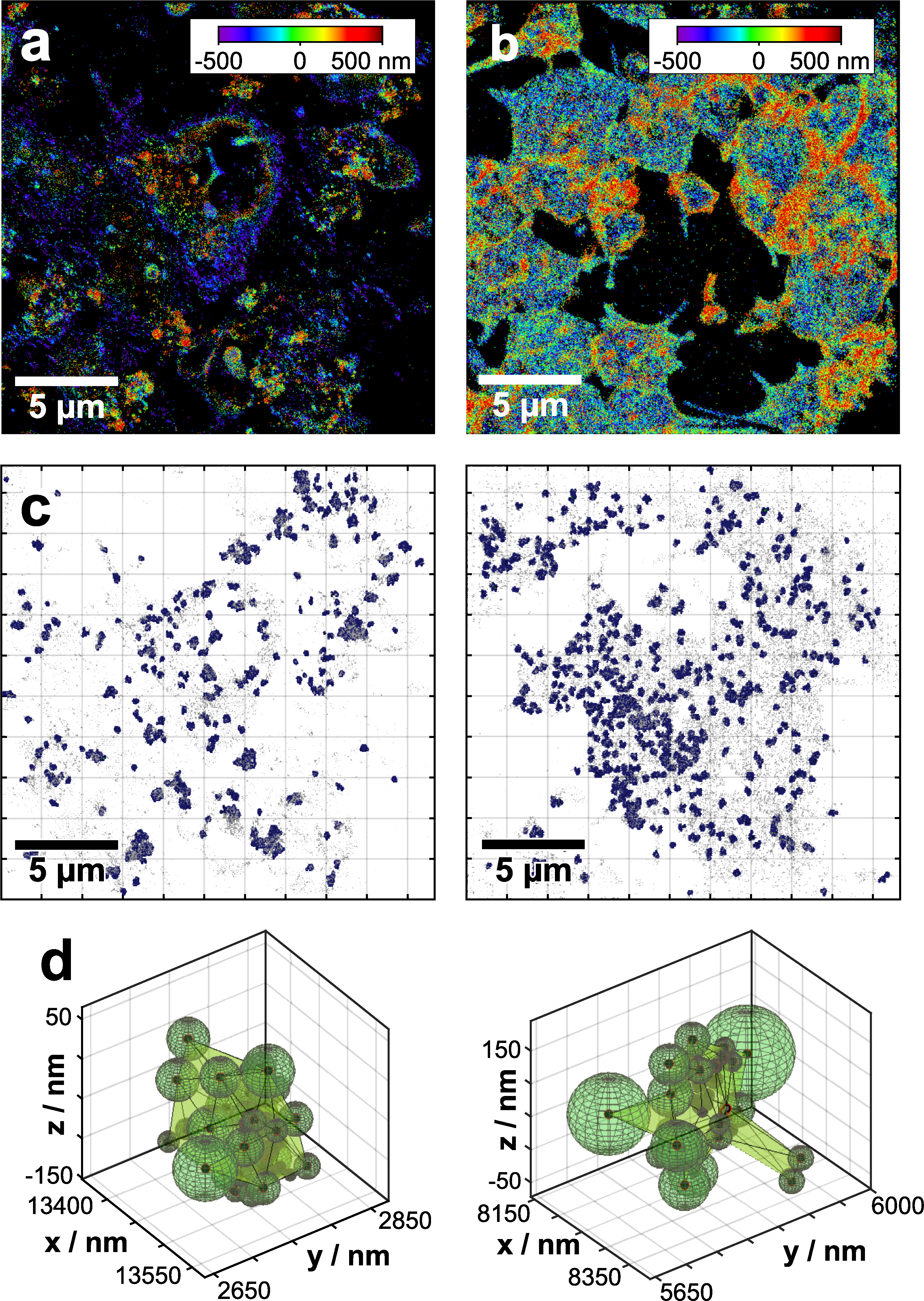

Supplement: S7 Fig — In one clot sample, CD41 was marked with an Alexa488-labelled antibody; in the other sample, CD62p was marked with an Alexa647-labelled antibody. We used our software platform to compare the two distributions of CD41 and CD62p molecules determined from the centres of two biological replicas of clots. Both reconstructed images are depicted in (a) and (b) (both reconstructed from approx. 300 000 localisation events). (c) shows the image of clusters with the cluster dimension of 235 nm (600 clusters in each image). For cluster representation, the Delaunay-triangulation method is used. (d) depicts two randomly chosen clusters. (TIF) [file pcbi.1007902.s008.tif]

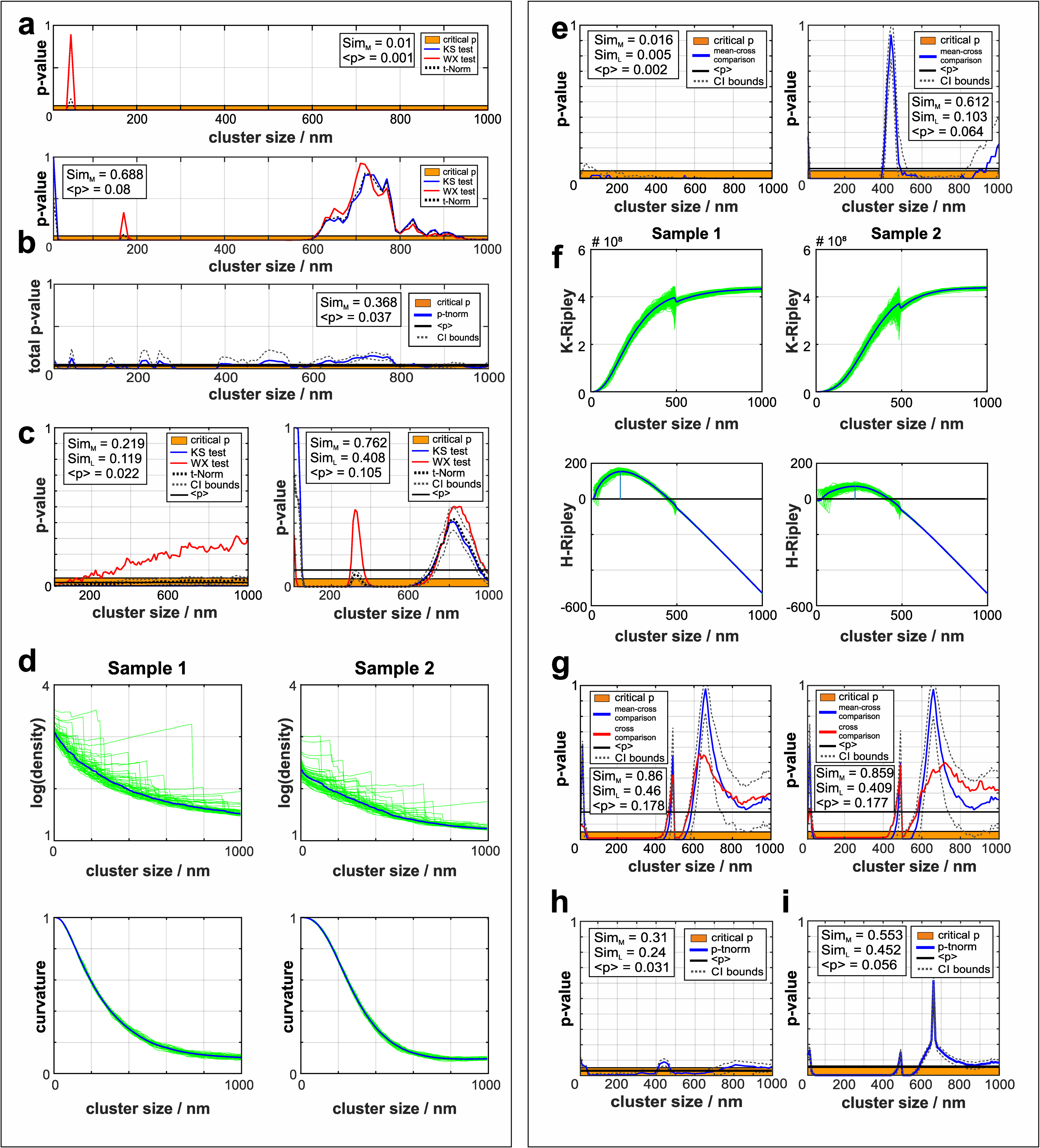

Supplement: S8 Fig — (a) shows the detailed analysis of the full samples. In this case, density (top) and curvature (bottom) distributions of all clusters for each cluster dimensions were compared at one time using a KS-/ WX-test (blue/red). The results indicate the dissimilarity of clusters for all cluster dimensions. As an indicator, an aggregated p-value of KS- and WX-test is determined using the weighting t-Norm-AND. The p-value (black dashed line) remains mostly below the critical p-value area (orange bar) disproving the similarity hypothesis. The corresponding values for (dis)similarity simM = 0.01/0.68 for the density and curvature distributions were determined respectively. A closer look at the KS-/WX-Test results on curvature comparison indicates that for cluster dimensions approx. 600 nm– 800 nm the distribution is above the critical p-value area (indicating similarity). (b) shows the aggregated p-value of density and curvature calculated with the weighting t-Norm-AND (blue) and the confidence interval (black dashed line). A simM = 0.36 is determined. The full-sample analysis was only, additionally performed, in order to show the full software capabilities (results are typically not used for a multilayer analysis). For such large samples, the fast bootstrap resampling is used. The graphs (c-d) clearly show a general dissimilarity of the samples. (d) depicts 100 curves–representing relative density of 100-resamples each including 2000 points (green)–used for the bootstrap based comparison of the cluster/curvature density distribution for both samples. In black, the averaged curve is represented. The detailed analysis of the bootstrapped samples (d) was performed as described in (a). (c) shows the statistical comparison of the cluster density (top) and curvature data (bottom), determined from the bootstrap results. For such detailed analysis (c), KS/WX- tests (blue/red) as well as the t-Norm-AND values (black) are plotted. Additionally, the boundaries of the confidence interval [file pcbi.1007902.s009.tif]

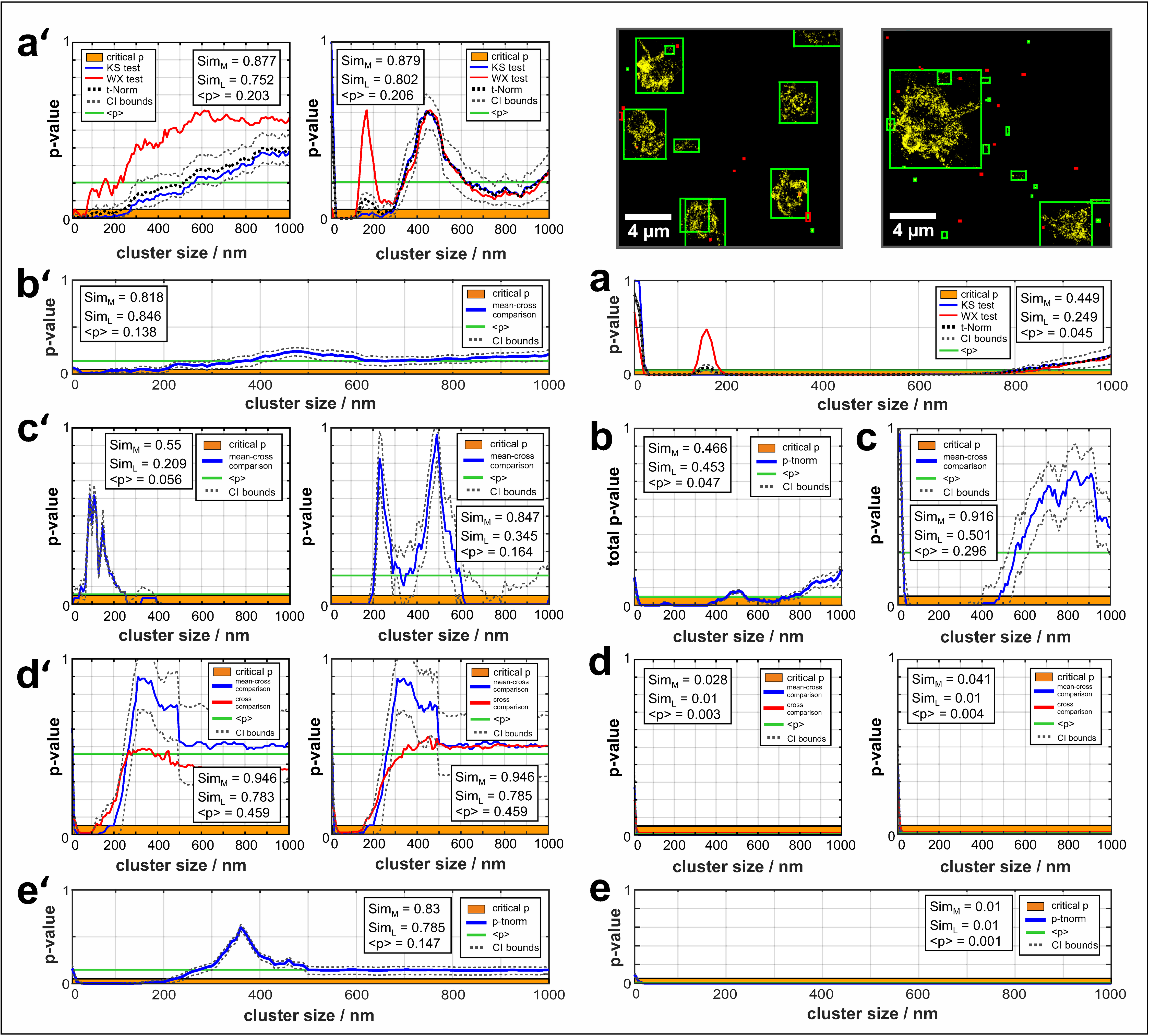

Supplement: S9 Fig — Cells were labelled with Alexa647-labelled anti-CD62p-antibody. Images were taken at the edge of an artificial clot, where platelets are more sparsely distributed than within the clot. Therefore, segmentation (ROI extraction) is required. Here, we present a statistical comparative analysis of the full image, with sparsely distributed platelets and we compare the extracted regions. The full (not regionalized) sample analysis is depicted in the left column of the Fig (a’-e’), the ROI analysis on the right ((a-e); parts of the ROI analysis are included in Fig 3 in the main text). From the analysis, the advantage of segmentation (region extraction) is clearly visible. The first level analysis of the comparison of the KS/WX- tests presented in (a) (and Fig 3E) and (a’) indicates a significant similarity for full images. For ROI-based comparison, density and curvature of the clusters shows similarity solely for cluster dimensions >700 nm (simM/simL = 0.47/0.31 and 0.45/0.24 for cluster density/curvature distributions, in contrast: simM/simL = 0.87/0.75 and 0.87/0.80 for a full sample). Fig 3C (main text) depicts two extracted regions of an untreated (upper) and IL-1β treated (lower) clot; a difference in the CD62p distribution can be observed. The divergence of the results between ROI and full image analysis is due to numerous small clusters, which are homogenously distributed in the images; in particular in the platelet-free area of the interleukin-1β treated clot. Not-cell-related signals add a significant additional cluster population, which is less significant within a ROI. For full image analysis, clusters for a specific cluster dimension originate from various regions due to hierarchical clustering, which influences the overall cluster density/curvature distribution. This is not the case for ROI-based analysis. Similar results can be observed for the second level analysis: the mean-cross comparison of the statistical data on cluster density and curvature show diverg [file pcbi.1007902.s010.tif]

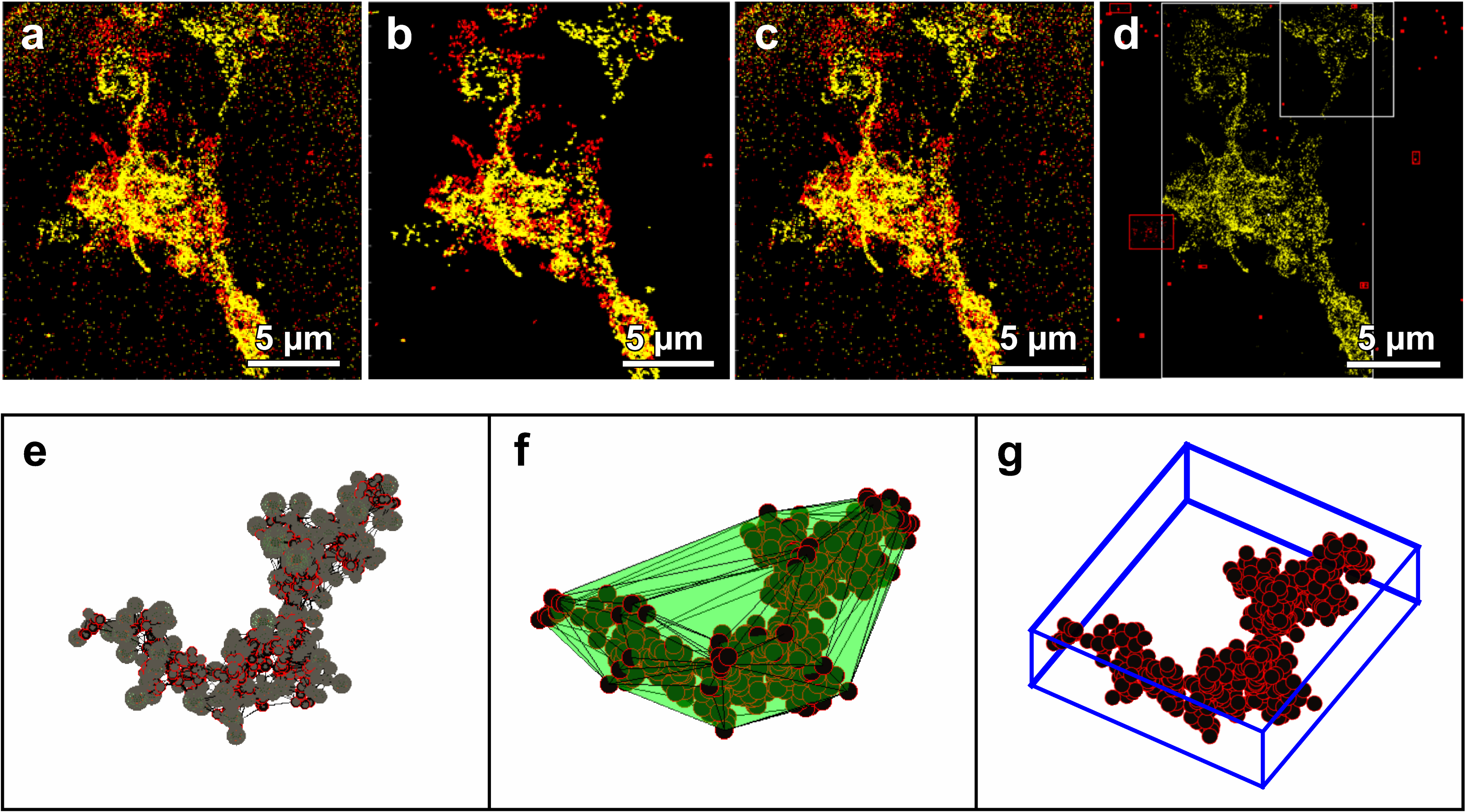

Supplement: S10 Fig — Images are reconstructed from approx. 60 000 single localisations. Images are shown before (a) and after (b) filtering using DBSCAN. Reduction from 59 393 points to 51 323 points (red: negative z values, yellow: positive z values) was achieved by using ρ = 2 and minP = 3. Points defined as outliers are filtered. (c) represents the sample image shown in (a) before region extraction (white box: large regions, red box: small regions). (d) shows the extracted region (region includes all points from original image). Cluster volume was calculated as sum of spheres (e), convex-hull volume (f) and box volume (g). (TIF) [file pcbi.1007902.s011.tif]

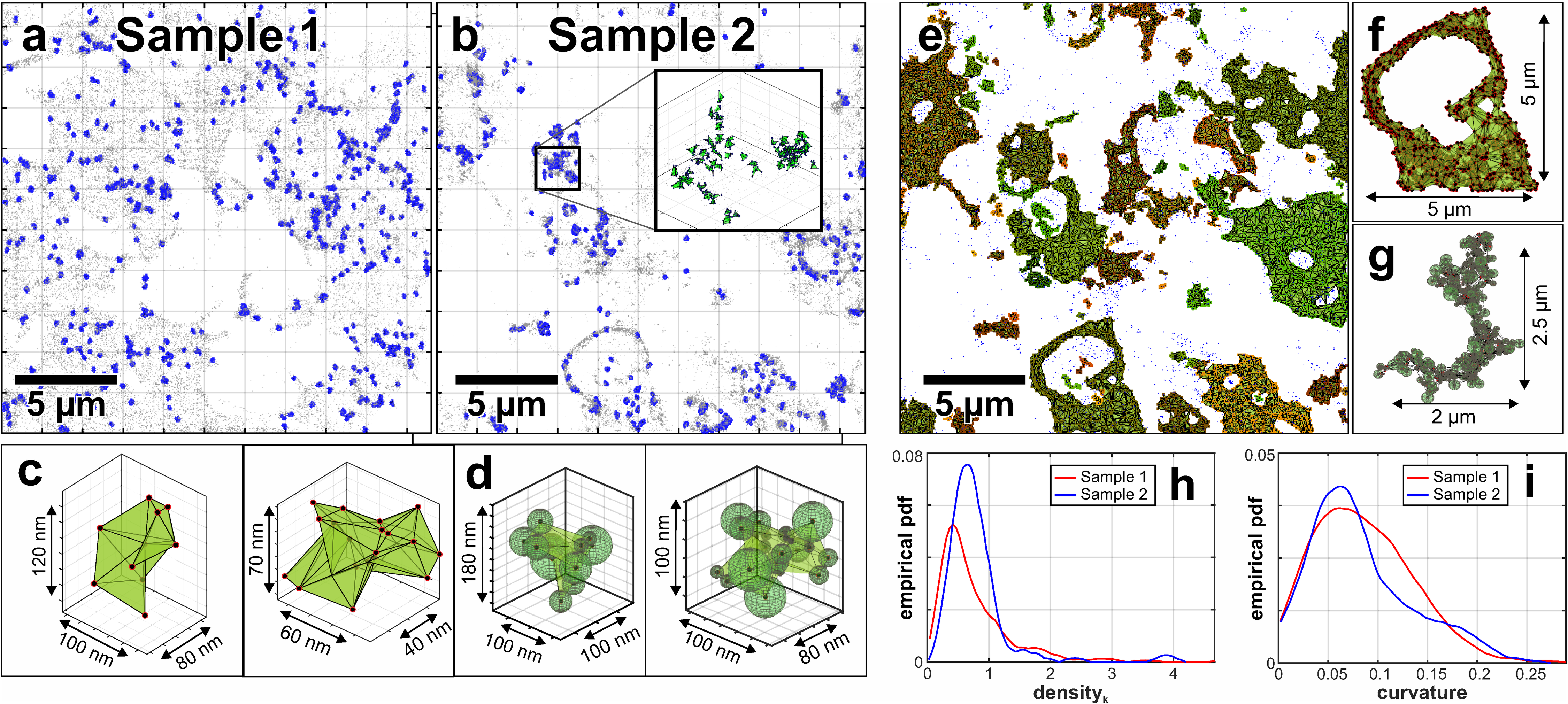

Supplement: S11 Fig — Comparison of different visualisation methods of clusters. Visualisation of clusters with a maximum size of 210 nm. (a) shows the 600 densest clusters of sample 1 and (b) the 600 densest clusters of sample 2. (c) depicts the triangulation-based representation of a cluster from the inset in (b). (d) shows the sphere packing-based representation of the chosen clusters. Visualisation of the primary clusters of sample 2, created with a neighbourhood radius of 250 nm. (e) depicts the largest primary clusters, (f) the triangulation-based representation of the largest cluster and (g) the sphere packing-based representation of the largest cluster. (h) shows the empirical probability density function (pdf) for density distribution of primary clusters. (i) shows the empirical pdf for curvature distribution of primary clusters. The red and blue curves in (h,i) represent the samples from (a) and (b), respectively. (TIF) [file pcbi.1007902.s012.tif]

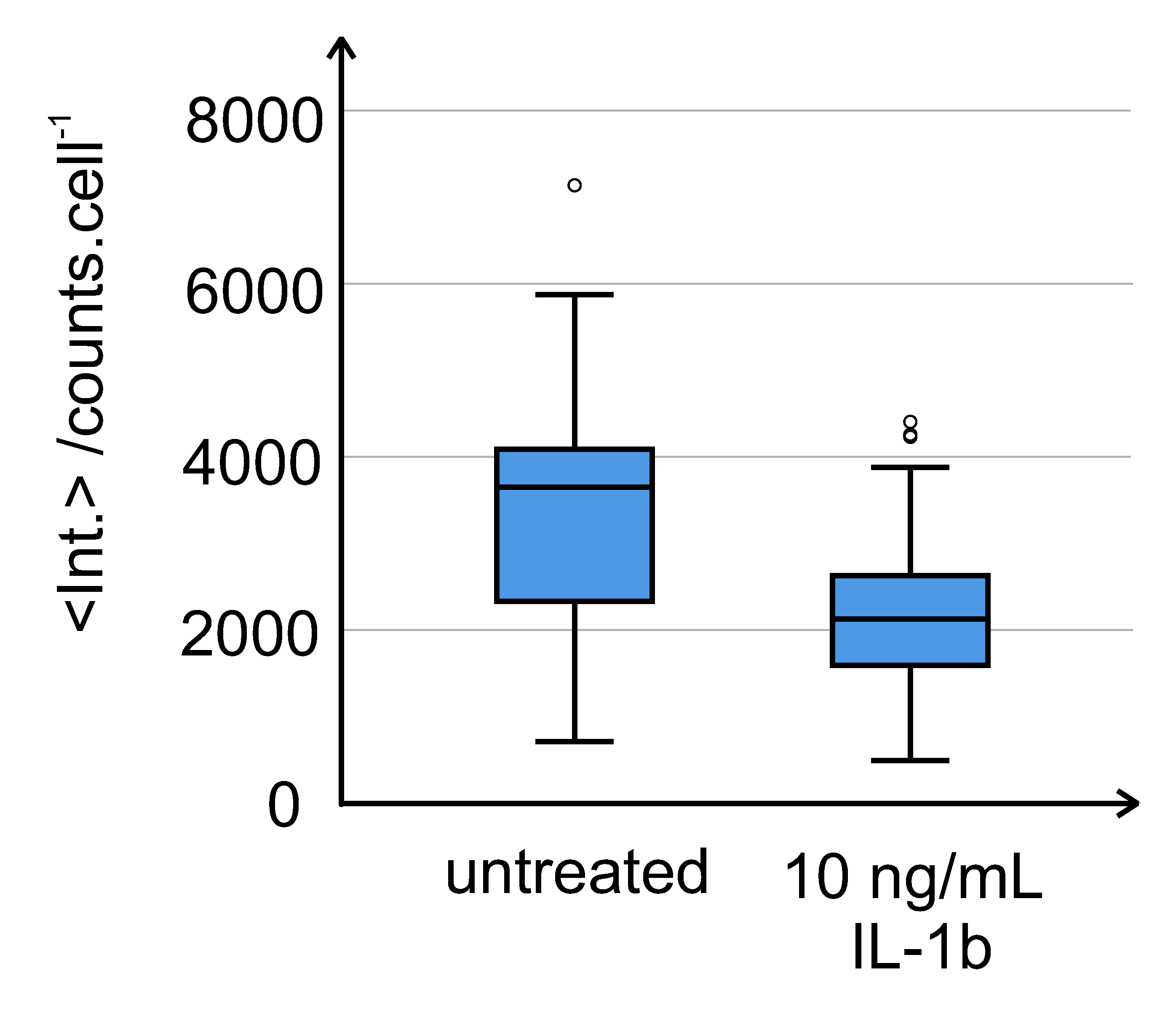

Supplement: S12 Fig — CD62p expression (detected as mean cell intensity (int.) of cells labelled with anti-CD62p-Alexa647) was significantly lower after 30 minutes of 10 ng/mL interleukin-1β treatment (mean: 5219 +- 1856 counts; n = 88 compared to 3565 +- 1246 counts; n = 83; power: 0.94). These data show a statistically significant lower CD62p expression for IL1-β treated cells in 2D (i.e. on single spread platelets). The boxplot depicts the median and the first and third quantile and outliers. (TIF) [file pcbi.1007902.s013.tif]

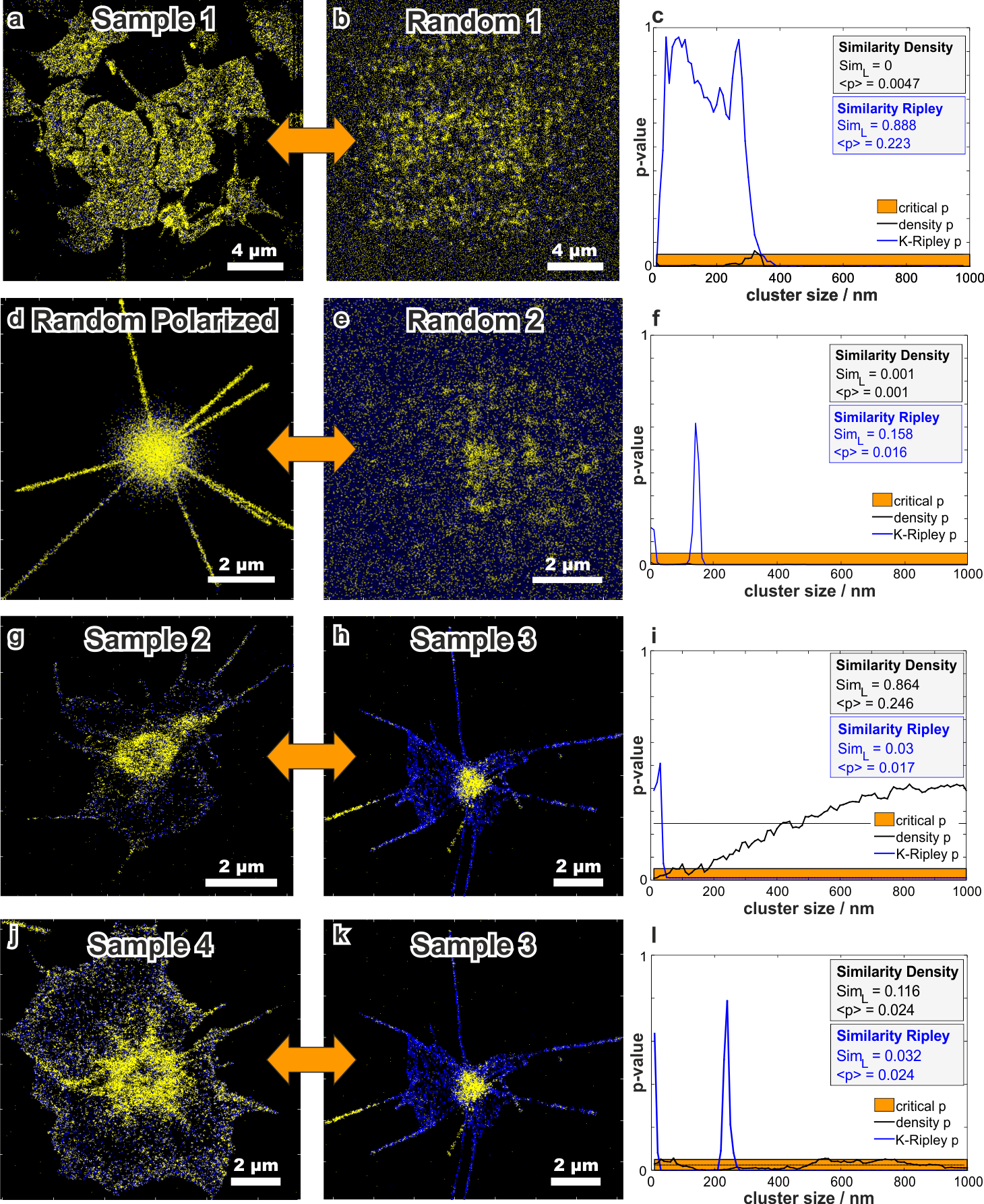

Supplement: S13 Fig — (a) shows the distribution of CD62p labeled with Alexa647-antibody. (b) represents a randomly distributed point cloud (approx. 85 000 data points/image). (c) shows the comparison of the cluster densities distribution to Ripley’s K-function distributions for all cluster dimensions between the simulated and CD62p datasets (black/blue line for cluster density/K-function, respectively). The lines depict the aggregated p-value of KS- and WX-test results. The test based on the Ripley’s K-function classifies the samples as similar (similar for cluster sizes ranging from 5 nm to about 400 nm). In contrary, our classification estimates the samples as dissimilar, with the test based on the density distribution of clusters (similarity measure = 0). The MLP neural network classifies samples as dissimilar with a posteriori probability of 88%. (d) and (e) show a simulated polarized (randomly distributed) and unpolarised image respectively. (f) shows the comparison of clusters density/curvature distributions and Ripley’s K-functions for all cluster dimensions between the simulated polarized and simulated unpolarized datasets (black/blue line for cluster density/K-function, respectively). The lines depict the aggregated p-value of KS- and WX-test results. The test based on the Ripley’s K-function classifies the samples as dissimilar (similar for cluster sizes ranging from approx. 150 nm– 180 nm). Our classification estimates the samples as dissimilar, with the test based on the density distribution of clusters (similarity measure = 0). Three additional pairs of polarized and unpolarised samples have been compared, rendering a dissimilarity of 78.5%, 88.5% and 95.1%. (g) and (h) show reconstructed dSTORM images of Alexa647-phalloidin labelled actin skeleton of two individual platelets activated on a glass surface. The two platelets are comparable, in an early activation state. (i) depicts the comparison of the cluster densities and the Ripley’s K-function distributions for all clust [file pcbi.1007902.s014.tif]

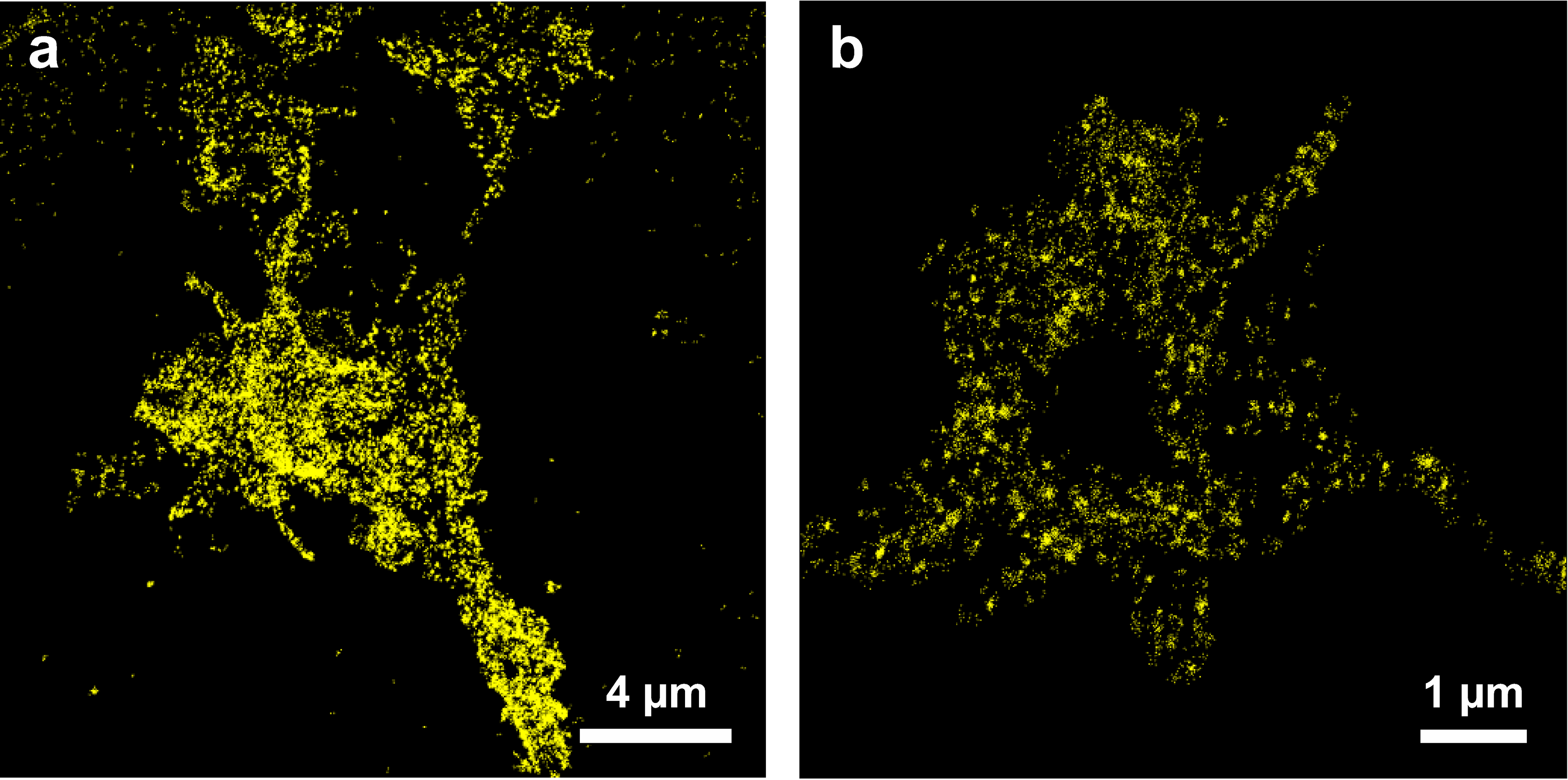

Supplement: S14 Fig — Comparison between the CD62p distribution in interleukin treated clots (large clot on the left side and a chosen region of interest on the right). First level statistics yields for the two samples similar cluster densities for all cluster dimensions (simM = 0.85) and for cluster curvatures (simM = 0.32). Similar values are obtained for a comparison of aggregated density and curvature values (simM = 0.76 and simM = 0.85). Dissimilarity for the H-/K-Ripley analysis can be observed (simM < 0.2). An MLP value of 0.66 proves the similarity of the samples. The detailed analysis directly shows the crucial dimensions and parameters, which best describe the differences between both samples. The results prove that the differences between large clusters as well as small ROIs (size ranges of individual cells) can be very precisely determined. (TIF) [file pcbi.1007902.s015.tif]
